# Accurate and reliable estimation of kinetic parameters for environmental engineering applications: A global, multi objective, Bayesian optimization approach

**DOI:** 10.1016/j.mex.2019.05.035

**Published:** 2019-06-07

**Authors:** Derek C. Manheim, Russell L. Detwiler

**Affiliations:** Department of Civil and Environmental Engineering, University of California Irvine, United States

**Keywords:** A Global, Multi Objective, Bayesian Optimization Approach for Parameter Estimation of Unstructured Kinetic Models, Parameter estimation, Model calibration, Unstructured kinetic models, Global optimization, Bioremediation, Water and wastewater treatment

## Abstract

Accurate and reliable predictions of bacterial growth and metabolism from unstructured kinetic models are critical to the proper operation and design of engineered biological treatment and remediation systems. As such, parameter estimation has progressed into a routine challenge in the field of Environmental Engineering. Among the main issues identified with parameter estimation, the model-data calibration approach is a crucial, yet an often overlooked and difficult optimization problem. Here, a novel and rigorous global, multi objective, and fully Bayesian optimization approach that overcomes challenges associated with multi-variate, sparse and noisy data, as well as highly non-linear model structures commonly encountered in Environmental Engineering practice is presented. This optimization approach allows an improved definition and targeting of the compromise solution space for all multivariate problems, allowing efficient convergence, and a Bayesian component to thoroughly explore parameter and model prediction uncertainty. This global optimization approach outperformed, in terms of parameter accuracy and precision, standard, local non-linear regression routines and overcomes issues associated with premature convergence and addresses overfitting of different variables in the calibration process.

• A sequential single, multi-objective, and Bayesian optimization workflow was developed to accurately and reliably estimate unstructured kinetic model parameters.
• The global, single objective approach defines the global optimum (the best compromise solution) and “extreme” parameter solutions for each variable, while the global, multi-objective approach confirms the “best” compromise solution space for the Bayesian search to target and convergence is assessed using the single objective results.
• The Approximate Bayesian Computational approach fully explores parameter and model prediction uncertainty targeting the compromise solution space previously identified.

## Graphical abstract

**Figure:**
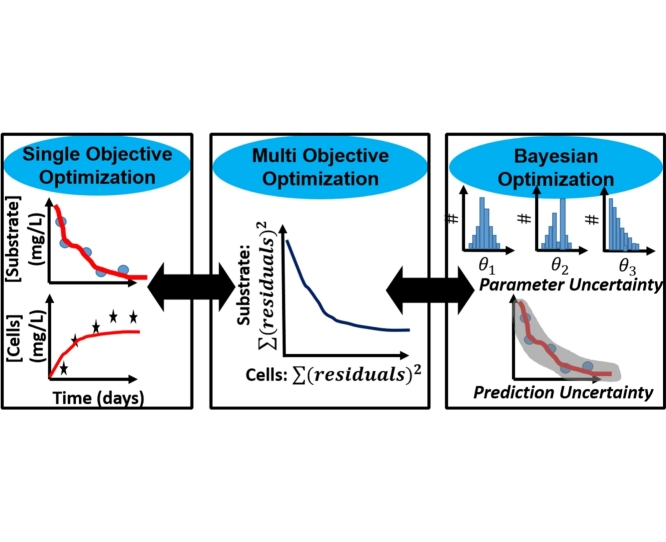

**Specifications Table**

**Table:** 

Subject area:	*Civil & Environmental Engineering*
More specific subject area:	*Bioremediation of Drinking Water Resources*
Method name:	*A Global, Multi Objective, Bayesian Optimization Approach for Parameter Estimation of Unstructured Kinetic Models*
Name and reference of original method:	AMALGAM-SO algorithm [28], DREAM-ZS algorithm [38], DREAM-ABC algorithm [39], NSGA-III algorithm [33]
Resource availability:	https://www.sciencedirect.com/science/article/pii/S0043135418309357*Links to dataset of fitting results.*

## Method details

### Introduction and background

Unstructured kinetic models, such as the well-known Monod model, have become widespread in the field of Environmental Engineering, ranging from air pollution control, water and wastewater treatment, and bioremediation to effectively describe and parameterize bacterial growth in engineered systems [[1], [2], [3], [4]]. These models provide a relatively simplistic, practical, and unified basis to predict microbial metabolism or transformation of nutrients, toxic chemicals, or production and synthesis of biochemicals throughout different media ranging from air, soil, and water [3,5]. Often, these models do not have a firm theoretical basis (as most were initially empirically derived) and holistically portray the cell, through various biokinetic parameters (i.e., the maximum specific growth rate, half saturation constant), as an enzyme “unit” that functions similar to the behavior described by different enzyme-kinetic models such as the Michaelis-Menten (Monod) or Hill (Moser) equations [1,6,7]. Despite these over-simplifications, unstructured kinetic models have reliably and accurately reproduced experimental data from all fields mentioned above and form the foundation for design and operational practice of biological based treatment and remediation systems [4].

**Fig. 1:**
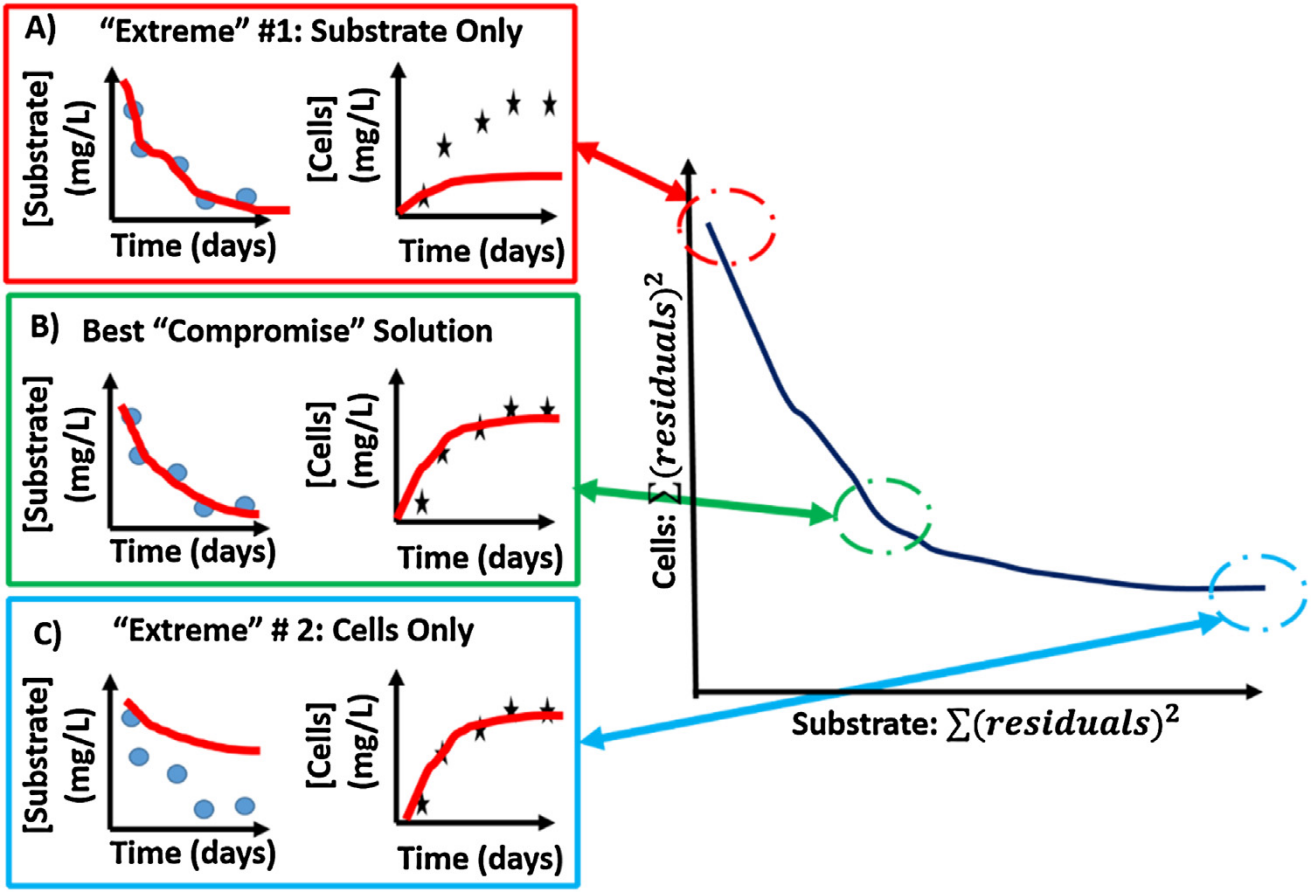
Graphical representation of the extreme solutions (A and C) as well as the compromise solutions (B) terminology referred to in this research method. The dark blue line in the righthand figure represents the compromise solution space.

To improve the overall accuracy and predictive utility of unstructured kinetic models describing biological treatment in the context of Environmental Engineering, a systematic approach must be undertaken, where model selection, parameter estimation (a.k.a., model identification or calibration), and model validation are critical steps underlying this approach (see [8], Fig. 1 for reference). Given some initial experimental data, modelling objective, and/or hypotheses about the treatment process, the model selection step makes a preliminary comparison of the accuracy and precision of several model structures (which differ in the mechanisms included that describe bacterial growth and substrate depletion) that are available to describe the treatment process [[8], [9], [10]]. It is important to note that depending on the overarching goal or objective of the model, this step could also entail building or modifying a new or existing mechanism into or within the framework of an existing model to account for new or different phenomena. Following this initial model selection or modification procedure, it is crucial to accurately and precisely identify and calibrate the parameters of the model. The parameter estimation step relies on several, inter-related components including: a) a parameter sensitivity analysis, to determine which parameters are most influential to the model output [11]; b), the optimal design of the experiment which may include a practical and structural identifiability analysis [12,13]; c), as well as the calibration procedure, which, in the case of most models encountered in the Environmental Engineering field, relies on a global, or perhaps local, non-linear regression routine [14]. Finally, given some data that was reserved from the data collection effort, the last step of this workflow will be to validate the accuracy and precision of the calibrated model against unseen experimental data (i.e., cross validation) [8].

Of the steps outlined in this systematic approach to improve the accuracy and reliability of unstructured kinetic models, the uncertainty associated with parameter estimates and model predictions and difficulties arising from non-linear regression for model calibration often challenge the application of these kinetic models in an environmental context, which is the focus of this developed method [2,[15], [16], [17], [18]]. Bayesian statistical techniques can offer insight into the uncertainty associated with model parameters and with the model structure itself (i.e., epistemic errors). Of the number of challenges identified, arriving at unique, accurate, and precise parameter estimates is a primary issue that often undermines the predictive utility of unstructured kinetic models. For example, reaching uncorrelated estimates of the maximum specific growth rate and half saturation constant of many unstructured kinetic models remains a well-known challenge [[19], [20], [21], [22]].

As introduced in the systematic approach to model implementation above, issues previously encountered with parameter estimation in bioremediation practice result from inadequacies in the experimental design, quality of experimental data collected, and the model-data calibration procedure [[23], [24], [25]]. The model-data calibration procedure is critical to obtain reliable parameter estimates and is often an overlooked, challenging non-convex optimization problem [14,18]. Generally, difficulties arise during model-data calibration including: 1) the experimental datasets analyzing biodegradation of pollutants are often multivariate, sparse, and noisy in nature; and 2) the unstructured kinetic models used to describe these datasets are highly non-linear [14,18,[26], [27], [28]]. In this study, we emphasize that multi-variate datasets present even more challenges, such as overfitting, where one variable may be given more weight during the calibration process.

It is perplexing that many past biodegradation studies [e.g., 5,18,29] have relied on deterministic, local nonlinear regression techniques for parameter estimation, as techniques based on gradient descent (like *FMINCON* provided by *MATLAB*’s optimization toolbox) may suffer from a lack of exploration of the search space and become trapped in local solutions. To overcome these exploration and convergence issues, stochastic, global optimization methods, including evolutionary algorithms (i.e., differential evolution), can be applied as robust solutions to this parameter estimation problem. Evolutionary algorithms (i.e., differential evolution), that are built on randomly evolving a population of individuals based on their fitness, are well known in the optimization field as effective and reliable global optimization approaches [30,31]. Although the application of these approaches in the field of bioremediation is still rather limited, several recent studies have applied variants of evolutionary algorithms, such as particle swarm, to investigate kinetic parameters describing the biodegradation of BTEX compounds [32]. In addition, several toolboxes have been developed in the literature for non-linear parameter estimation of biological models that include both local and global search capabilities, including the AMIGO series [33,34]. Although these toolboxes provide reliable optimization algorithms, they do not offer a fully Bayesian, likelihood free approach to evaluate the parameter and model predictive uncertainties.

In this research method, we describe a novel and rigorous approach to accurately and reliably estimate parameters in unstructured kinetic models given multi-variate experimental datasets based on a sequential global, single objective, multi objective, and fully Bayesian optimization procedure. In the following section (A global, multi objective, and Bayesian optimization approach to parameter estimation), we give an overview of the workflow behind our approach, introduce key elements of the unstructured kinetic models and datasets used for model-data fitting comparison, and provide an in-depth description of the methods involved for improved parameter estimation. In the final section (The case for global optimization: research method validation), we demonstrate the utility of this research method by comparing the performance of the algorithms used in this optimization approach to local, non-linear regression methods.

### A global, multi objective, and Bayesian optimization approach to parameter estimation

**Fig. 2:**
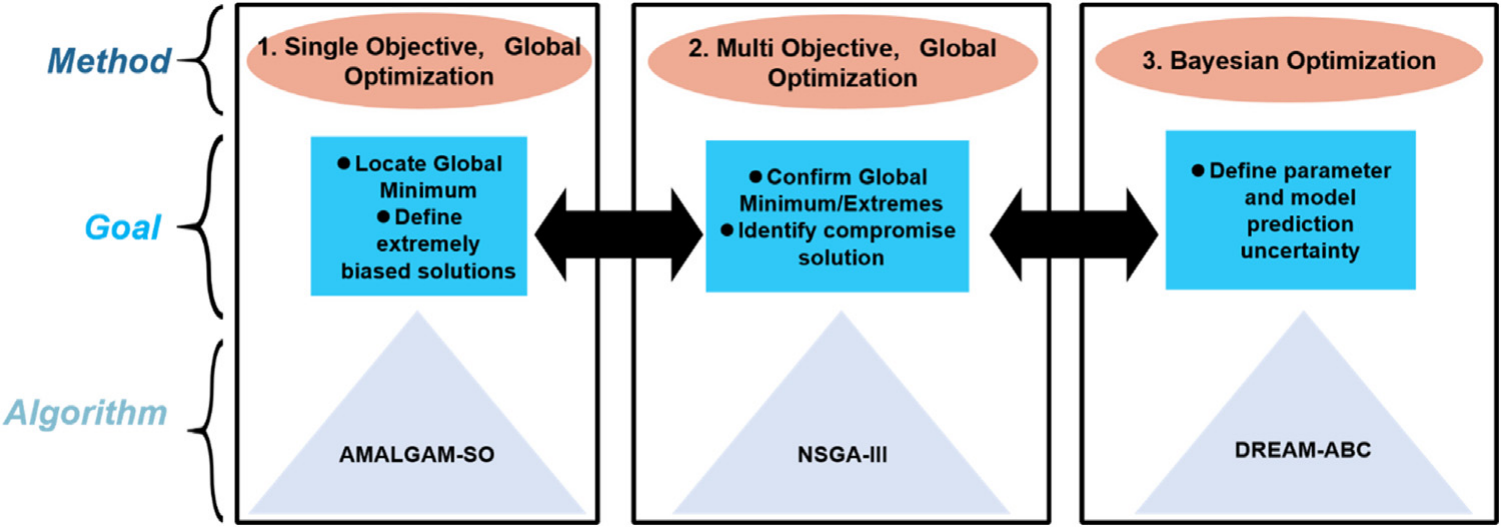
The primary optimization methods, goals, and algorithms used in this study for parameter estimation.

The main workflow for this research method is detailed in Fig. 2, which portrays a sequential approach to improved parameter estimation, including the following three steps: Step 1) a single objective, stochastic optimization algorithm locates the global optimum (i.e., the best compromise solution) and the “extreme” solutions; Step 2) a multi-objective, stochastic optimization algorithm targets the best compromise solution using results from the previous, single objective step to verify proper convergence of the multi-objective approach, and; Step 3) an Approximate Bayesian Computational (DREAM-ABC) approach develops a posterior distribution in parameters using the verified “best” compromise solution to target the correct compromise solution space around the global minimum. These steps are reversible in the sense that the current step should be verified or rely on information from a previous step of the workflow (Fig. 2). Step 2 of this workflow provides further certainty that the compromise solution space has truly been reached (providing necessary redundancy and robustness), as two different optimization frameworks (single vs. multi) will have converged to the same area of the search space. Although initially three SO and MO algorithms were applied in this approach, we recommend that only the best performing algorithms listed in Fig. 2 are necessary for proper convergence and parameter estimation.

The most critical improvement this workflow brings is: a) an improved definition and targeting of the compromise solution space for multi-variate calibration problems to avoid overfitting of different variables and b) the Bayesian component to explore parameter and model prediction uncertainty. Here, the compromise solution space is designated as the set of solutions (that are centered around the global optimum or the best compromise solution) that represent the optimized tradeoffs between different objective functions (Fig. 1). The global optimum is equivalent to the best compromise solution of the compromise solution space, located at the solution (in the objective function space) closest to the nadir or apex of a curve formed between the set of compromise solutions (Fig. 1). The extreme solutions, contrarily, are found when one variable, such as cell or substrate concentration, is fitted at a time as opposed to simultaneously (Fig. 1A and C). The extreme solutions exist at the beginning and end of the curve that passes through the compromise solution set (Fig. 1).

#### Overview of unstructured kinetic models and datasets for model-data fitting comparisons

In this study, parameter estimation was performed for a variety of unstructured kinetic growth models describing microcystin (MC, a cyanobacterial toxin present in drinking water) biodegradation by isolated, homogenous bacterial populations [10]. Each of these models describes the specific growth rate of degrading bacterial cells (*μ*) as a non-linear, positively increasing function of substrate concentration (*S*) (where interested readers are referred to [10] for a complete description of the mathematical formulations and parameters included). As an example, the well-known Monod model describes the specific growth rate of bacterial cells as a hyperbolic function of the substrate concentration (Eq. (1)). The maximum specific growth rate, half saturation constant, and the yield coefficient (*μ*_*max*_, *K_s_*, and *Y*) describe the maximal growth rate of a bacterial population when the substrate is non-limiting, the bacterial population’s relative affinity for a specific substrate, and the yield of new bacterial biomass per substrate consumed [35]. Other unstructured kinetic models, such as the Moser model, possess a similar mathematical framework to the Monod model, but incorporate additional parameters (such as *S*^*n*^, where *n* is an additional model parameter) to describe other important underlying physical processes [36].

(1) $$\mu =\frac{\mu_{\text{m}\text{a}\text{x}}S}{K_{s}+S}$$

During a batch biodegradation experiment (i.e., where MC is the sole limiting carbon source, aerobic, temperature/pH controlled), the time dependent change in substrate concentration (*S*) and bacterial degrading biomass (*X*) can be described by the following coupled set of ordinary differential equations (Eqs. (2) and (3)), where the endogenous decay of bacterial cells during growth is explicitly considered [37,38]:

(2) $$\frac{dS}{dt}=-\frac{1}{Y}\mu X$$

(3) $$\frac{dX}{dt}=\mu X-k_{d}X$$

Where *S* is the limiting substrate concentration (mg/L), *X* is the biomass concentration (mg/L), *μ* is the specific growth rate of bacterial cells (1/h), *Y* is the cell yield coefficient (unitless), and *k_d_* is the endogenous decay coefficient (1/h). Importantly, *X* = b*C, where *b* is a linear scaling factor used to convert optical density or cell concentration data (*C*) into biomass concentrations (*X*).

Experimental datasets for model-data fitting comparisons performed in this study were acquired from four different studies evaluating MC biodegradation of isolated, homogenous bacterial populations [[39], [40], [41], [42]]. These studies isolated bacterial populations from the *Sphingomonas* (designated Study 3, [39]), *Sphingopyxis* (designated Studies 2 and 4, [40,41]), and *Bacillus* genera (designated Study 1, [42]), which are representative of the main populations involved in MC degradation [[43], [44], [45]]. In accordance with the assumptions introduced above for Eqs. (2) and (3), each of these studies performed batch degradation experiments, where the initial bacterial inoculum, MC concentrations, temperature, pH, and the availability of oxygen and nutrients were tightly controlled (see [10] for a complete description and comparison). Across all experiments, both the substrate (MC) and bacterial biomass concentrations were quantified daily for a 1.3-10-day time period using HPLC (for MC) and optical density (OD 600 nm) or plate counts (for biomass) as general quantification methods.

**Fig. 3:**
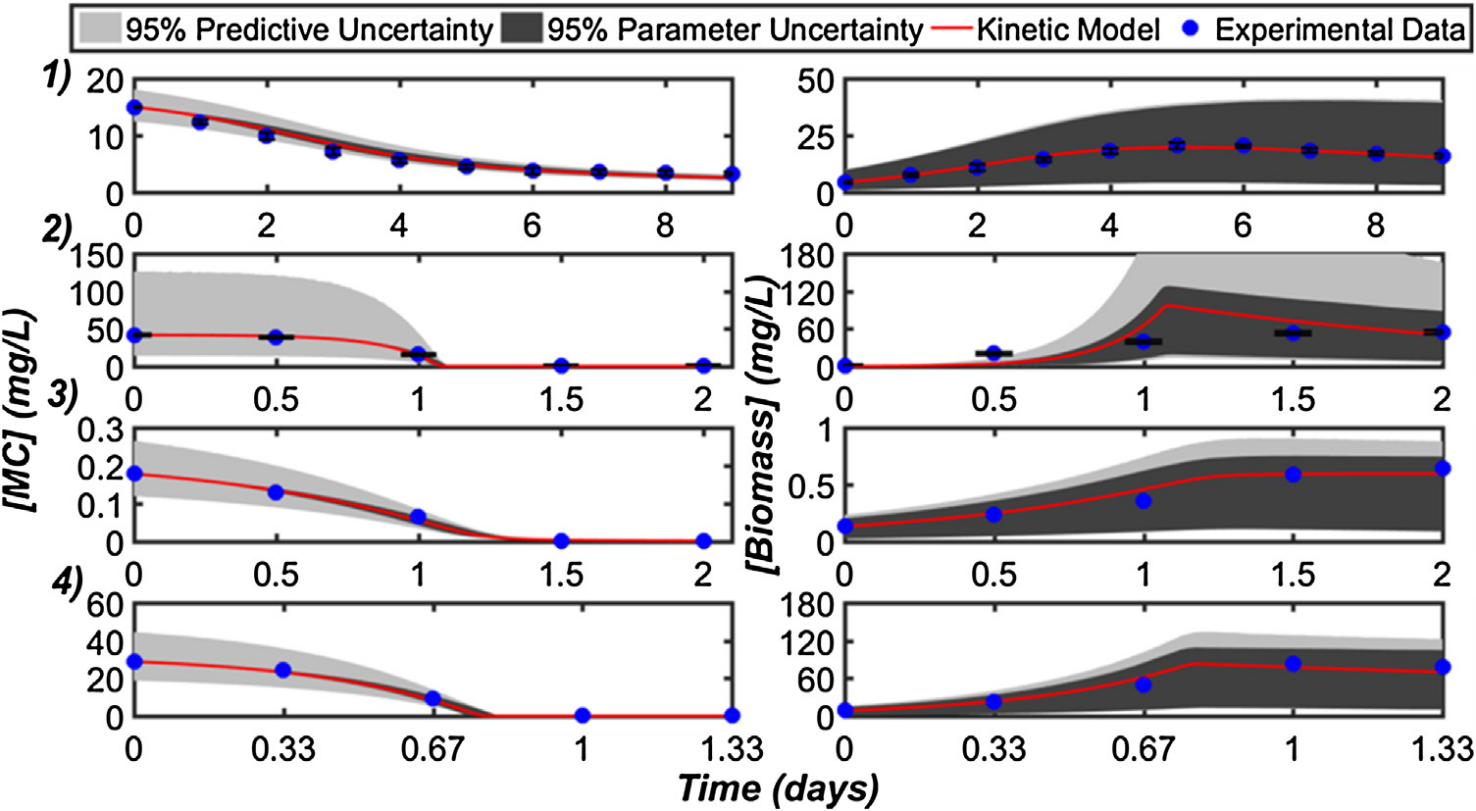
Kinetic model-experimental data fitting results of the best performing model for Studies 1–4 portraying MC removal (first column) and corresponding biomass growth (second column). The red line indicates the best fitting model prediction, while the blue dots represent the experimental data points (along with the standard deviation of replicate experiments). The light grey shading indicates the 95% predictive uncertainty interval and the dark grey shading represents the 95% uncertainty interval associated with the parameter estimation.

As an example, Fig. 3 highlights the experimental results obtained from each study overlaid with the best fitting unstructured kinetic models determined from a Bayesian model comparison and selection process [10]. Studies 1–3 were fit using the Moser model, whereas Study 4 was fit using the Heijnen model [46]. As observed in Fig. 3, all experimental data points were observed to fall within the uncertainty intervals, indicating that the predictions afforded by either model can reproduce the experimental data with great certainty. Overall, the relatively few time points for data collection and some data points with moderate variability (i.e., Study 2) were reflective of the general sparse and noisy nature of many datasets associated with batch biodegradation experiments.

#### Global, single objective optimization (GSO) approach

The GSO approach involved the application of an extensively tested series of SO evolutionary optimization algorithms, including Self-Adaptive Differential Evolution (saDE) [47], the multi-algorithm evolutionary based AMALGAM-SO optimizer [48], and an advanced variant of the LSHADE (cnEpSin) series of algorithms [49]. The saDE algorithm was directly coded in MATLAB (*MATHWORKS*, Inc., r2015b) based on the description presented in Qin et al. [47], whereas MATLAB production codes were acquired for the AMALGAM-SO and LSHADE-cnEpSin algorithms. Exact details of the mechanism used behind each optimization algorithm are presented elsewhere (see [[47], [48], [49]] for specific information). Although only one algorithm is generally sufficient for use in future optimization problems, we observed some utility in benchmarking at least three different stochastic approaches to ensure that the global minimum was in fact reached. If the user is considering selecting only one of these algorithms, we recommend running multiple independent repetitions (changing the initial random seed), to ensure that the optimizer consistently reaches the global minimum solution.

Prior to applying each algorithm to the experimental datasets acquired, fifteen of the CEC 2005 benchmark functions were used to rigorously test and compare the optimization performance of each GSO. From this initial testing period, the AMALGAM-SO algorithm demonstrated the most reliable performance, followed by the LSHADE (cnEpSin) and saDE algorithms, respectively (Supplementary information, Section 1). In addition, optimal run conditions and control settings for each algorithm were identified based on this prior testing and were kept consistent when applied to the experimental data acquired herein.

The formal Gaussian Log-Likelihood function was used as the primary objective function for GSO and GMO optimization approaches, similar to the approach presented by Knightes and Peters [18,29]. In this study, we assumed that the error residuals for each variable were independent, normally distributed (with zero mean), and exhibited constant variance (homoscedastic). A formal check of these three main assumptions is presented in the Supplementary information, Section 2 of this article for reference. Of the three primary assumptions regarding the error residuals, homoscedasticity was difficult to fully justify given the small sample sizes of each experimental dataset [50]. Because the formal tests for heteroscedasticity (i.e., White’s, Engle’s, Breusch and Pagan’s) applied in this study rely on some form of regression, ultimately, a greater number of datapoints will be necessary to make any reliable statistical inferences of the trends in the error residuals as a function of the measured variables. It is likely that the variance of the experimental measurements is proportional to the measurement signal, leading to some inherent heteroscedasticity among the error residuals [51,52]. Nevertheless, the number of experimental replicates for each study was relatively small (<3), which precluded a full and reliable justification of the homoscedasticity of the error residuals.

In addition, we assumed that the covariance between the variables (i.e., cell and substrate concentration) was negligible; however, this assumption may not be entirely realistic as past studies have indicated a range in slightly positive to moderately negative correlations between substrate (log-transformed) and biomass (not log-transformed) measurements during PAH biodegradation [18]. The statistical confidence that the covariance between these measurements was non-zero ranged from 33% to 94%, demonstrating that there is some inherent value to explicitly accounting for covariance during the optimization process [18]. In this study, we argue that log transformation of both the substrate and biomass measurements, placing them on equivalent scales, may, to some extent, reduce the correlation between measurements. Moreover, the relatively small number of data points (5–10) may undermine statistical hypothesis testing that would validate the inclusion of covariance during the optimization procedure. Furthermore, this assumption not only simplified the objective function calculation but provided more reliable parameter estimates compared to using a form of the objective function that considers covariance between the dependent variables (data not shown). The Gaussian Log-Likelihood objective function (*OF_1_*) to minimize thus reduces to a function of the sum of square residuals (*SSR_1_* and *SSR_2_*) and the overall standard deviation for each variable (σ_Y1_ and σ_Y2_) after making these necessary simplifying assumptions (Eq. (4)) [18,53].

(4) $${OF}_{1}=\frac{1}{{\sigma_{Y1}}^{2}}\;({SSR}_{1})+\frac{1}{{\sigma_{Y2}}^{2}}\;({SSR}_{2})$$

Although standard deviations were given in each dataset for each observation, we decided to fit each model using the average of replicate experiments, given that the number of repetitions was low for each study (three or less) [28]. This assumption also eliminated the requirement for including the overall standard deviations of each variable as weight in Eq. (4) above. To reduce bias related to the magnitude of the model predictions, the logarithm (base 10) of model predictions was used in calculation of the objective function values [18,29].

##### GSO run conditions and control settings

Standard run conditions were set for each SO algorithm to ensure fair performance for each model-dataset calibration. These run conditions included a fixed number of function evaluations (500,000) and equivalent termination criteria. The termination criteria for saDE and LSHADE- cnEpSin were dependent on three criteria: a) exceeding the maximum function evaluations; b) meeting the following tolerance: if the range of the objective function values of the population members was less than 1E-08; c) or meeting the following tolerance: if the range of the parameter values of all population members was less than 1E-02. Termination and restart criteria for AMALGAM-SO was identical to that described in [48]. However, global termination criteria were introduced so that if successive runs resulted in similar objective function values meeting a predefined tolerance, the search was stopped. Specifications of the exact run conditions and control settings used in each of these algorithms are specified in the Supplementary information, Section 1.1.

#### Global, multiple objective (GMO) optimization approach

The GMO approach involved the use of three different evolutionary algorithms including the improved NSGA-III genetic based algorithm [54], the multi-algorithm, multi objective AMALGAM optimizer [55], and the RVEA algorithm (RVEA) [56]. These MO algorithms were selected based on a formal comparison of at least ten different MO algorithms benchmarked on a suite of well-known MO test functions (Supplementary information, Section 3). The PLATEMO test platform was incorporated in this MO test comparison as a useful tool for benchmarking different algorithms [57]. Results of a formal benchmarking on standard, MO test functions indicated that the NSGA-III algorithm performed the best of the initial algorithms screened using a range of selection criteria (i.e., accuracy and convergence, diversity, and number of non-dominated solutions), followed by the AMALGAM-MO and RVEA algorithms (Supplementary information, Section 3). Similar to the SO approach, optimal run conditions and control settings for each MO algorithm were identified and kept consistent when applied to the experimental data acquired herein.

##### GMO run conditions and control settings

Standard run conditions were set for each MO algorithm to ensure fair performance for each model-dataset calibration. These run conditions included a fixed number of generations (20,000) to run each MO algorithm, which was determined by successively running an increasing number of generations until the change in the non-dominated solution sets was deemed negligible (after 5 independent repetitions) (data not shown). In addition, the population size was fixed to N = 100 for each algorithm. The AMALGAM-MO, NSGA-III and RVEA algorithms were run with identical control settings as specified in the Supplementary information, Section 3.1. It is important to note that the NSGA-III and RVEA algorithms were run using the *MATLAB* code developed by the PLATEMO user interface [57].

#### Bayesian optimization approach

A posterior distribution in parameter estimates was reached through the DREAM-ZS (Differential Evolution Adaptive Metropolis, sampling from past states) (v3.0) software package [58]. Unlike the previous approaches, we chose a likelihood free method using Approximate Bayesian Computation (ABC) to specifically target and facilitate convergence to the compromise region of the search space [59]. The specific objective function (*OF_2_*) incorporated in this study to maximize was similar to that presented by Sagdeh and Vrugt [59], which is based on the distance between the observed and predicted summary statistics (*m*) and some predefined tolerance, $\varepsilon_{j}$ (Eq. (5)). Importantly, the sum of squared residuals (for both cell and substrate data) from the compromise solution obtained by the best performing GMO were chosen as the observed summary statistics (*S_j_,* Eq. (5)) to guide the ABC method. In this approach, the sum of squared residuals (L2 norm) obtained during the ABC optimization procedure (for fitting both cells and substrate) represented the simulated summary statistics ($\hat{S_{j}})$.

(5) $${OF}_{2}={\text{m}\text{i}\text{n}}_{j=1:m}\{\varepsilon_{j}-\rho \left(S_{j},\hat{S_{j}}\right)\}$$

Where $\rho \left(S_{j},\hat{S_{j}}\right)$ simply represents the distance between the observed and simulated summary statistics: abs($S_{j}-\hat{S_{j}})$. The specific DREAM-ZS run conditions and control settings applied for the ABC algorithm are summarized in Table S10 for reference (Supplementary information, Section 4).

**Fig. 4:**
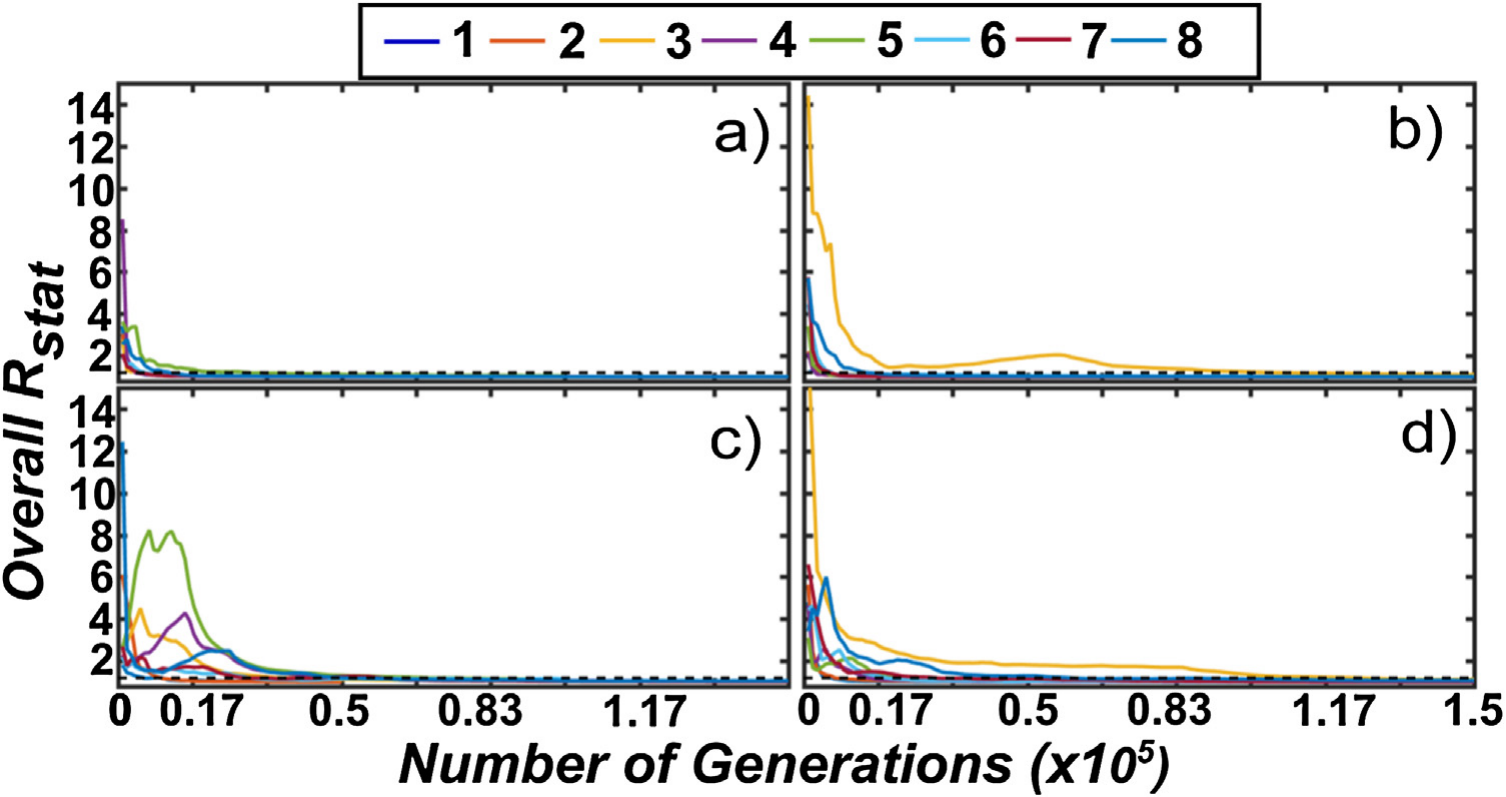
Evolution of the overall Gelman and Rubin R-statistic for the DREAM-ZS (ABC) algorithm when applied to models 1–8 for each corresponding dataset. The letters a–d correspond to Studies 1–4 and numbers 1–8 correspond to the Monod, Tessier, Contois, Blackman, Dabes, Powell, Moser, and Heijnen model structures. The dashed line indicates the convergence threshold of 1.2.

The overall convergence statistic of Rubin and Gelman [60] was summarized for each Study (1–4 identified in Section 2.1) to verify that the DREAM-ZS -ABC algorithm was running through enough generations to reach a stable estimate of the posterior distribution in parameters (Fig. 4). Convergence was assessed over a wide range in model structures to obtain insight into the performance of the ABC algorithm against various non-linearities, including (1–8) the Monod kinetic model [35], Tessier kinetic model [61], Contois kinetic model [62], Blackman kinetic model [63], Dabes kinetic model [64], Powell kinetic model [65], Moser kinetic model [66], and the Heijnen and Romein kinetic model [46].

For all studies and models investigated (a–d, 1–8), convergence was generally reached after 150,000 generations (corresponding to 900 K overall for 6 chains), where the R-statistics converge to a stable value below the 1.2 threshold for each model structure reviewed (Fig. 4). Across all studies, the Contois model demonstrated the largest number of necessary generations until convergence was reached, due to the more complex non-linear structure of the model (Fig. 4). In some cases (Study 4), the Dabes kinetic model also demonstrated poor convergence using the ABC method. These results indicated that reliable posterior distributions in parameters have been achieved after approximately 150,000–200,000 generations.

### The case for global optimization: research method validation

Finally, we highlight the importance of global optimization techniques to provide accurate and robust parameter estimates for all nonlinear regression problems as compared to standard, localized optimization methods. We expect that the global optimization approaches adopted in this study can handle the difficulty of fitting multivariate, sparse, and noisy data by avoiding local optima and locating compromise solutions that avoid overfitting one variable in a multivariate dataset. Here, the results of the *FMINCON* constrained nonlinear optimization tool provided by *MATLAB* (and commonly used for parameter estimation in past studies) are compared to the best performing global optimization algorithms used in this study. We limit this comparison of the optimization algorithms to parameter estimation of the Moser model structure, a common unstructured kinetic growth model, for experimental data detailing MC biodegradation and cell growth from a variety of laboratory studies introduced in Section 2.1 [[39], [40], [41], [42]]. The Moser model structure contains six parameters to be calibrated, including the maximum specific growth rate (*μ*_*max*_), the half saturation constant (*K_s_*), the yield coefficient (*Y*), the first order endogenous decay rate (*k*_d_), a linear cell concentration to biomass conversion parameter (*b*), and the exponent parameter (*n*) in the Moser model (Eqs. (2), (3), (6)). For each approach and Study (defined as 1–4, as indicated in Section 2.1), five independent repetitions were used to evaluate the mean and variance in the parameter estimates and objective function values.

(6) $$\mu =\frac{\mu_{\text{m}\text{a}\text{x}}S^{n}}{K_{s}+S^{n}}$$

The constraint settings for the *FMINCON* method in *MATLAB* were kept as equivalent to those used for the global optimization algorithms to ensure impartiality. For all studies, identical bounds used in the global optimization algorithms were applied to the *FMINCON* approach for constraining the feasible parameter space, where all other constraints were set to null values. Furthermore, the *FMINCON* approach used identical experimental datasets to those used for the global optimization routines, and the search was commenced using randomly initialized (uniformly distributed) starting points within the feasible parameter space. As recommended by *MATHWORKS*, the “interior-point” optimization approach was implemented in *FMINCON* as this algorithm has demonstrated success on both large, sparse problems as well as small, dense optimization problems [67]. To provide some insight into the performance of other available algorithms, the “active set” optimization setting was also selected in this comparison [68]. All the run settings for each algorithm (i.e., interior-point or active set) were kept at default values except for the stopping criteria. The stopping criteria were defined as follows: stop if a) the number of function evaluations was exceeded (500,000); b) the number of iterations was exceeded (500); c) the function tolerance (value of the objective function) was reached (1E-20); d) the step tolerance was reached (1E-20); or e) the constraint tolerance was reached (1E-20). It is important to note that the number of iterations (comparable to generations for the global optimization algorithms) was set to 500, which is considerably lower than the number stipulated for most global approaches, as most *FMINCON* searches stagnated within this window and performing additional iterations was deemed unnecessary. Moreover, the magnitude of the tolerances was set to very low values to ensure that the *FMINCON* search avoided premature convergence.

As demonstrated in Table 1, different parameter estimates were obtained between the *FMINCON* and best performing SO, global optimization algorithms. The *FMINCON* approaches resulted in larger variation in parameter estimates as compared to the global optimization approaches (Table 1). This variation in parameter estimates is most likely due to the nonlinear solver in *FMINCON* becoming stuck in local optimal solutions and prematurely converging. In most cases using the *FMINCON* algorithms, the search was terminated due to the step size tolerance threshold, indicating that these methods encountered difficulty thoroughly exploring and exploiting the search space. These local optimization methods were also sensitive to the initial values the parameters were set to, where some repetitions demonstrated considerable improvement over others. The global optimization algorithms consistently reached the same global optimum, as confirmed by the low standard deviation of all parameter estimates (Table 1). These results imply that the global optimization methods, despite their stochasticity, are robust nonlinear regression techniques, where reliable parameter estimates can usually be achieved with high probability.

**Table 1:** Parameter estimates for FMINCON and best performing global, single objective optimization algorithms. The mean and standard deviation are presented for each parameter and approach.

Study	Approach	μ_max_	K_s_	Y	K_d_	b	n
1	FMINCON- Active Fit	15.8	155	3.08	1.16E-01	1.79E-01	9.89
		13.9	52.3	4.71E-01	1.81E-01	3.57E-01	2.11 E-01
	FMINCON- Interior Point	18.60	152	2.52	4.44E-02	2.89E-02	9.84
		7.0	76.2	5.65E-01	2.02E-02	2.62E-02	1.38 E-01
	AMALGAM-SO	1.08	220	2.15	1.28E-01	8.06E-03	2.19
		6.53E-03	1.05E-02	1.39	8.00E-04	5.23E-03	6.40E-03

2	FMINCON- Active Fit	13.38	71.0	2.93	4.23E-01	8.61E-02	6.28
		9.6	47.9	1.07	4.81E-01	7.70E-02	2.96
	FMINCON- Interior Point	8.30	104	2.12	4.68E-01	7.65E-02	5.41
		4.28	65.1	5.13E-01	2.33E-01	2.40E-02	1.41
	AMALGAM-SO	6.23	1.21E-01	2.49	8.09E-01	5.98E-02	9.61
		1.80E-01	5.07E-02	1.44	1.23E-01	3.62E-02	5.92E-01

3	FMINCON- Active Fit	19.9	120	0.86	1.00E-02	2.68E-08	2.47
		16.0	63.8	1.42	5.16E-05	4.13E-08	3.02
	FMINCON- Interior Point	11.9	40.9	2.16	1.00E-02	4.15E-08	9.09E-01
		12.3	52.8	1.80	4.72E-07	5.34E-08	1.29E-01
	saDE	2.06	4.78E-02	2.90	3.38E-02	1.31E-10	1.59
		9.32E-01	6.38E-02	4.08E-01	3.81E-02	2.38E-11	5.39E-01

4	FMINCON- Active Fit	16.4	48.2	1.45	2.28E-01	5.82E-01	3.37
		20.8	83.9	1.38	3.05E-01	8.17E-01	3.76
	FMINCON- Interior Point	13.0	64.3	1.90	2.60E-01	7.56E-01	5.88
		19.9	75.6	1.16	2.40E-01	7.10E-01	3.43
	AMALGAM-SO	2.89	1.80E-04	2.69	9.35E-02	5.09E-01	2.59
		1.68E-01	2.23E-04	1.37	1.22E-01	2.65E-01	1.12E-01

In terms of predictive accuracy, the global optimization methods returned the smallest mean objective function (total sum of squared residuals) values and largest Log-Likelihood values (data not shown), with small standard deviations observed in general (Table 2). The FMINCON optimization methods, contrarily, suffered from poor predictive accuracy, as mean objective function values were larger than those obtained by the global optimization approaches (Table 2).

**Table 2:** Objective function estimates for FMINCON and best performing global, single objective optimization algorithms. The mean and standard deviation are presented for each parameter and approach.

Study	Approach	MinOF	Subs %	Cells %
1	FMINCON-Active Fit	7.61	88	12
		4.17	12	12
	FMINCON- Interior Point	8.61	96	4
		8.86E-01	1	1
	AMALGAM-SO	3.29E-02	88	12
		1.96E-05	0	0

2	FMINCON- Active Fit	5.12	73	27
		3.78	29	29
	FMINCON- Interior Point	1.88	58	42
		1.87	35	35
	AMALGAM-SO	5.97E-01	16	84
		1.04E-03	0	0

3	FMINCON- Active Fit	3.01	46	54
		2.74	23	23
	FMINCON- Interior Point	1.53	29	71
		4.74E-02	3	3
	saDE	2.17E-01	88	12
		1.26E-01	5	5

4	FMINCON- Active Fit	6.14	85	15
		11.2	14	14
	FMINCON- Interior Point	15.2	86	14
		13.8	15	15
	AMALGAM-SO	4.12E-02	88	12
		8.12E-03	6	6

The ability of the SO algorithms to reach the best compromise solution was further compared using the percent contribution of substrate and cell concentration fitting error to the overall fitting error (% Subs or % Cells) (Table 2). This analysis is simply dissecting Eq. (4) presented above (without standard deviations of the measurements included, σ_Y1_ and σ_Y2_) into a contribution to the overall objective function (*OF_1_*) from fitting either the cell concentration or MC substrate experimental data. Here, we benchmark the SO algorithms with the solution obtained using the MO approach. The MO global optimization results indicated the following percent contribution (% Subs/Cells) for the best compromise solution for each Study (1–4): 87.5/12.5; 15.4/84.6; 82.9/17.1; 90.3/9.7. It is important to note that the best compromise solutions do not result in a proportional tradeoff (i.e., 50/50%) between fitting the cell and substrate concentration data. Instead, for most Studies (1,3,4) the compromise solutions showed a better fit to the cell concentration as compared to the substrate concentration data, as the cell data indicated a higher contribution to the overall objective function.

The results demonstrated that for Studies 2 and 3, the compromise solutions reached were different than the compromise solutions defined above for the *FMINCON* algorithms. However, although the accuracy was not high, the relative tradeoff between fitting the substrate vs. cell concentration data was similar to that obtained by the multi objective approach for studies 1 and 4 using the *FMINCON* algorithms (Table 2). As expected, the SO global optimization approaches provided comparable compromise solutions to those obtained by the multi objective optimization approaches.

Since a strong dependence of the local optimization methods on the initialization location in the search space was observed, we investigated whether the performance would be enhanced if the initialization was set very close to the global optimum solution. For this run of experiments, we narrowed the search space of the local optimization methods to an arbitrarily small hypercube (i.e., six-dimensional space) around the global optimum solution (see Supplementary information, Section 5). The parameters were still randomly initialized in this smaller subspace using uniform random sampling and each local optimization method was run using identical settings as described above. Again, for each approach and Study (1–4), five independent repetitions were used to evaluate the mean and variance in the parameter estimates and objective function values.

Even within a very close vicinity to the global optimum solution, the results showed that the local search methods prematurely converged to a local solution located around the global optimum (Table 3). This result is evident as the magnitude of most parameter values are not the same when comparing the best performing global optimization and the *FMINCON* optimization results for each study (Table 3). Although the local optimization results of the constrained test cases still indicated that the global solution was not reached, the performance of the *FMINCON* algorithms was far superior to the case where the search boundaries were less constrained (Tables 1 vs. 3).

**Table 3:** Parameter estimates for FMINCON and best performing global, single objective optimization algorithms when the search space was constrained. The mean and standard deviation are presented for each parameter and local optimization method. The best parameter set achieved for the global optimization methods after 5 repetitions are presented for reference.

Study	Approach	μ_max_	K_s_	Y	K_d_	b	n
1	FMINCON- Active Fit	1.0776	219.511	2.740	0.12884	0.0102	2.189
		2.05E-03	3.64E-01	4.02E-02	6.80E-04	1.34E-04	6.44E-04
	FMINCON- Interior Point	1.0720	219.578	2.723	0.12461	0.0103	2.183
		5.48E-05	5.00E-03	8.67E-04	4.72E-05	2.87E-06	2.31E-04
	AMALGAM-SO	1.0784	220.000	2.796	0.12852	0.0104	2.189

2	FMINCON- Active Fit	6.301	0.0896	3.304	0.8611	0.0767	9.551
		1.01E-01	9.93E-04	2.17E-01	5.19E-02	1.95E-03	2.69E-01
	FMINCON- Interior Point	6.370	0.0810	3.453	0.8657	0.0760	9.630
		8.40E-02	7.94E-04	1.73E-01	9.21E-03	1.80E-03	1.89E-01
	AMALGAM-SO	6.357	0.0822	3.451	0.8981	0.0780	10.000

3	FMINCON- Active Fit	1.235	0.000100	1.777	0.0125	7.80E-11	2.268
		1.55E-02	0	2.82E-02	3.39E-03	2.74E-12	1.48E-03
	FMINCON- Interior Point	1.227	0.000100	1.782	0.0100	7.90E-11	2.268
		3.42E-03	1.39E-15	4.06E-02	2.54E-06	2.24E-12	1.45E-04
	saDE	1.231	0.000100	1.740	0.0100	7.66E-11	2.268

4	FMINCON- Active Fit	2.771	0.000101	3.341	0.0240	0.6440	2.615
		4.12E-02	2.97E-06	4.28E-02	5.26E-03	3.92E-02	8.67E-03
	FMINCON- Interior Point	2.756	0.000104	3.353	0.0241	0.6618	2.614
		3.23E-02	4.73E-06	2.70E-02	3.88E-03	3.36E-02	4.74E-03
	AMALGAM-SO	2.789	0.000100	3.351	0.0295	0.6380	2.617

In addition, there was a noticeable difference in performance between the two variants of the *FMINCON* algorithms, when comparing the mean absolute error calculated between the optimal parameter set (out of five repetitions) from the best performing global and local optimization parameter estimates and the standard deviation of parameter estimates. In general, the performance (benchmarked using the mean absolute error) using the *FMINCON* Interior Point method was improved over the Active Set method for Studies 1 and 2, whereas the Active Fit method was better than the Interior Point method for Studies 3 and 4 (data not shown). Moreover, the Interior Point method demonstrated a less variable performance compared to the Active Fit method, as the standard deviation of most parameters using the Interior Point method were lower than those obtained using the Active Fit method (Table 3). These results confirm that even when the search is constrained further, local optimization methods may still not equipped with the adequate tools to thoroughly explore the feasible search space as compared to global optimization methods. Similarly, the performance of local search routines was observed to be sensitive to the gradient descent method selected as well as the control settings specified for optimization.

**Fig. 5:**
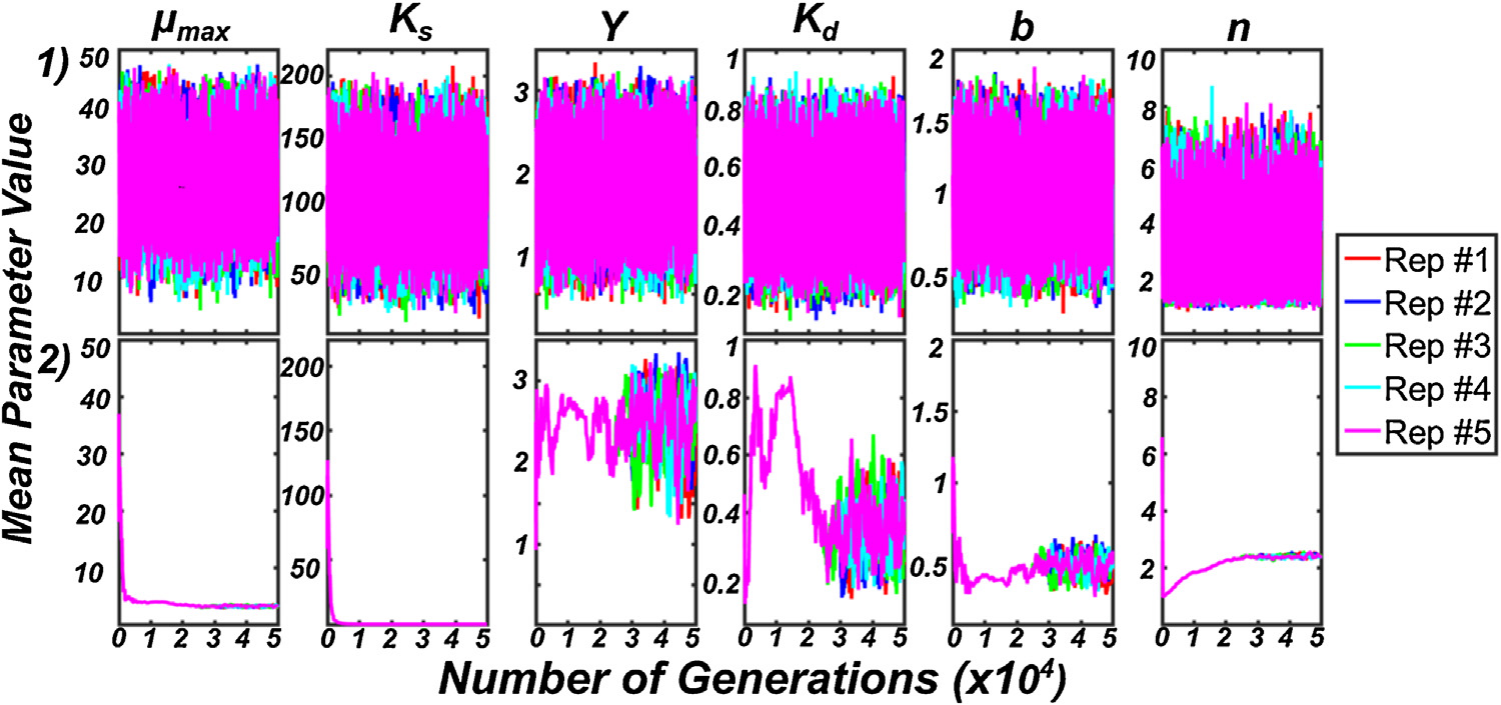
Evolution of the mean (across all Markov chains) parameter values for both the 1) DREAM-ZS (Gaussian Likelihood) and 2) the DREAM-ZS (ABC) algorithms when calibrated against the Moser model using the fourth experimental dataset. The results of five independent repetitions are presented, as differentiated by the color scale of the legend.

Another significant improvement achieved from this research method workflow resulted from the integration of Likelihood free Bayesian optimization approaches (i.e., Approximate Bayesian Computation) as a final step, which allowed an enhanced targeting of the compromise solution space as compared to formal Gaussian Likelihood approaches. To demonstrate the benefits of likelihood free approaches, we briefly compared the convergence and parameter identifiability of the Bayesian optimization methods using both Likelihood free and formal Gaussian Likelihood methods. The simplest form of the Gaussian Log-Likelihood function was used (similar to Eq. (4)) and corresponded to option # 11 presented in Table 2 of the DREAM software package theory and implementation (Vrugt [69]). The Moser model structure was used to compare both methods, as previous results (Fig. 4) demonstrated that a higher number of generations were required to reach convergence when calibrating this model structure using the ABC approach (for most studies). All the control settings for both approaches were kept identical as described in Table S10. Similar to previous testing, five independent repetitions were used for each study/model combination to assess the variability in performance between the two Bayesian optimization approaches. Although the formal convergence efficiency (using the overall R statistic of [50]) was not significantly improved using the DREAM-ABC approach (Fig. S5), the parameter identifiability was enhanced for all studies (Fig. 5). As observed in Fig. 5, which presents the mean of all parameter values across each Markov chain at each generation, the DREAM-ABC algorithm gradually converged to a stable posterior parameter distribution after approximately 50,000 generations, while the posterior parameter distribution obtained using the Gaussian Likelihood method was highly variable (Fig. 5). This enhanced performance may be partly attributed to the inability of the DREAM approach to account for multiple objective functions using the built-in objective functions available. Although the user is free to create a custom objective function, there is no way to explicitly optimize two or more objective functions simultaneously. Similar improvements in parameter identifiability using the DREAM-ABC approach were observed using the experimental data from all other Studies (Supplementary information, Section 7). Thus, despite the greater computational effort required to define the compromise solution space using this new approach (i.e., Steps 1–2 of this workflow), ultimately, more reliable as well as meaningful parameter and model prediction uncertainty estimates can be achieved.

Although accurate and reliable parameter estimates were achieved in this study, it is important to address several inherent limitations to this approach. First, as observed in Table 2, this approach does not completely solve the overfitting issue. For a majority of the datasets, the compromise solutions were more biased towards fitting of the substrate as opposed to cell concentrations (˜80% vs. ˜20%). In addition, the only method used in this study to explicitly balance fitting between either variable was to re-scale each of the variables (by taking the log_10_) when calculating the objective function values (as the number of experimental replications was too few to effectively weigh each objective function by the standard deviation of measurements, [28]). It is likely that this calibration process, favoring estimation of the substrate concentration, may result in poor predictive performance of bacterial growth during the model validation step of the model implementation workflow. To equally weigh the objective functions from each fitted variable, it may be necessary to perform objective function normalization and/or introduce a subjective weighting term into Eq. (4) above. Promising normalization techniques may involve dividing each objective function by the best “extreme” solution or using the expected range in objective function values (i.e., min/max) to normalize the objective function values to the range [0,1] [70]. Once on an identical scale of analysis (i.e., [0,1]), a subjective weighting term of 0.5 can be applied to ensure equal weights during optimization [71]. This proposed weighting/normalization scheme, along with a model validation procedure, will be explored in future research to assess the effects of overfitting on the predictive capacity of unstructured kinetic models in the field of Environmental Engineering.

Another limitation of this approach was the computational burden associated with the parameter estimates. As a succession of single, multi-objective, and Bayesian optimization algorithms were applied, the computational load far exceeded that of the local, non-linear methods. Although the evaluation of each objective function was relatively quick using MATLAB’s ODE solvers, the large number of function evaluations required for each generation (up to 200,000 generations for the Bayesian algorithm) of the evolutionary algorithms applied was sometimes overbearing when running on a three-core processor. We found that this approach works very well on a high-performance computing cluster where the function evaluations for each generation can be run in parallel on individual cores. Moreover, we expect that the computational burden will increase significantly as the number of model parameters and variables in each dataset increases. However, the computational burden can be significantly reduced if the multi-objective step is omitted from the workflow and only the best compromise solution is determined for the single-objective optimization algorithm. Although, omitting this second step will likely lead to some loss of certainty in the optimization procedure, it will greatly facilitate the parameter estimation process if the computational load is too overwhelming.

Although the comparisons made in this study demonstrated that the global optimization method resulted in improved parameter estimates as compared to the local, non-linear solver, we stress that these results should not completely rule out the application of local methods for parameter identification of unstructured kinetic models. The comparison made in this study for the local optimization algorithm was generally informed by default options, standard algorithms, and tolerance settings from MATLAB’s tutorials concerning *FMINCON*, not from ample user experience of the “best” optimization routine to take for a given non-linear model and dataset. For example, other non-linear regression routines available in MATLAB, including *LSQNONLIN* or *FMINSEARCH* (or combination thereof), which are not based on gradient descent, may be more efficient for the identification of parameters of unstructured kinetic models commonly used in Environmental Engineering applications. An alternative approach, in which an optimized non-linear routine is used instead of the standard nonlinear search routine used here, may lead to improvements in parameter estimation. For example, the local optimization routine could be optimized through application of combined *FMINCON* searches over different initial conditions (i.e., setting up a lattice as in [18]), where the minimum of this local search pattern would be defined as the global minimum. Application, standardization, and comparison of a more refined approach to local, non-linear regression to the global approach developed herein is a topic warranting future study.

Overall, given the observed differences in parameter precision, accuracy and bias observed between both approaches (i.e., local vs. global), the choice of optimization method is imperative to arrive at reliable parameter estimates for unstructured kinetic models describing biodegradation. In this study, we have proved that global optimization approaches offer some inherent advantages to nonlinear regression routines provided by *MATLAB*’s optimization toolbox. Advantages of this optimization method workflow include the ability to handle multi-variate datasets, which often present problems with overfitting of certain variables in addition to Bayesian estimates of the parameter and model predictive uncertainties. As evidenced above, global optimization approaches arrived at the best compromise solutions with higher probability than local methods. This method workflow also allowed a thorough review of both parameter and model prediction uncertainty through integration of an ABC approach. Ultimately, the Bayesian component of this method was found to be a powerful diagnostic tool for model comparison and selection purposes commonly encountered in the Environmental Engineering field.

## Funding

This work was supported by NSF CBET grant no. 1806066.
