# Supplementary material for: Accurate and reliable estimation of kinetic parameters for environmental engineering applications: A global, multi objective, Bayesian optimization approach
Source: MethodsX. 2019 Jun 7;6:1398–414. doi: 10.1016/j.mex.2019.05.035 (PMC6582191; doi:10.1016/j.mex.2019.05.035)
Supplement: Supplementary file 1 [file mmc1.docx]

**Accurate and Reliable Estimation of Kinetic Parameters for Environmental Engineering Applications: A Global, Multi Objective, Bayesian Optimization Approach**

**Supplementary Information**

Number of Pages: 21
Number of Figures: 8
Number of Tables: 11

Derek C. Manheim,^*1^ and Russell Detwiler^1^

^1^Department of Civil and Environmental Engineering, University of California, Irvine, CA, USA

^*^Corresponding Author: Department of Civil and Environmental Engineering, University of California, Irvine, CA 92617, email: [dmanheim@uci.edu](mailto:dmanheim@uci.edu); Tel: 858-334-8757

**1.0 Formal Comparison of Single Objective Optimization Algorithms**

Several genetic, evolutionary algorithms were benchmarked against 15 standard test functions to evaluate and compare their inherent optimization performance. This comparison was deemed necessary before testing took place on actual data sets as some algorithms (saDE) were coded from scratch and the performance of AMALGAM-SO and LSHADE-cnEpSin was relatively unknown. The standard test functions for benchmarking included unimodal, multimodal, and hybrid composition functions clearly defined in Suganthan et al. [*1*] and referred to by Vrugt et al. [*2*]. The following optimization algorithms were assessed in detail: classical differential evolution (DE) [*3*]; self-adaptive differential evolution (saDE) [*4, 5*]; AMALGAM-SO, a multi-method evolutionary single objective optimization algorithm [*6*]; and LHSHADE-cnEpSin, an enhanced adaptive evolutionary algorithm [*7-10*].

**1.1 Run Conditions and Control Settings for Single Objective Algorithms**

Specifications of the exact run conditions and control parameters used in each of these algorithms are specified below. AMALGAM-SO was run using the following three specified algorithms: CMA-ES, PSO, and GA, based on results presented in [*11*]. The control settings for each algorithm (i.e., PSO and GA) are identical to those presented in Table 3 of [*11*]. Similarly, the number of population members in AMALGAM-SO was set to evolve from 10, 20, 40, 80, 160, 320.

saDE was run using a learning period value of 20 generations and a median initialized cross-over value of 0.5 (with standard deviation of 0.1), which was the only parameter that was adapted, as specified by [*12*]. The following five mutation strategies were used in our version of saDE: DE/rand/1/bin; DE/rand2best/2/bin; DE/rand/2/bin; DE/current2rand/1; and original DE as defined in [*13*]. Stochastic universal selection (SUS) sampling was used to select the mutation strategies and the control parameters lambda and *F_de_* were selected from a normal random distribution (using a mean of 0.5 and standard deviation of 0.3) as specified by [*12*]. The number of population members per generation was fixed to 50 for each optimization run for the saDE algorithm.

The LSHADE-cnEpSin algorithm was run using mostly identical settings to those specified in [*14*]. Initialized values of the adapted scaling parameter (*F_de_*), crossover probability (*CR*), and the frequency of the sinusoidal search function were all set to 0.5. The memory size for adaptive storage and learning period, as specified in [*14*] was set to 5 and 20, respectively. The mutation strategy for LSHADE-cnEpSin was set to current2pbest/1, as used in [*14, 15*]. The probability of performing crossover using covariance matrix adaptation (*p_c_*) and the proportion of individuals used to generate the covariance matrix was set to 0.4 and 0.5, respectively, based on results presented in [*14*]. The initial size of the population and the minimum size of the population (after population size reduction) were set to 50 and 4, respectively. All other control settings were identical to those reviewed in [*14*].

**1.2 Testing Procedure for Single Objective Algorithms**

The testing procedure involved running the specified optimization algorithm on each individual test function for a total of 25 individual runs, where the random number generator was offset for each run to ensure that performance was varied for each run. All the problems were solved in D=10 dimensions, which was sufficient compared to the number of parameters used in realistic model simulations presented in this study. A predefined tolerance limit, as specified by [*1*] was used to monitor the convergence of each run. The maximum number of function evaluations was set to 100,000, which was of similar order of magnitude to that previously specified [*1*]. The total number of function evaluations required to reach the specified tolerance was recorded along with the minimum function value for each run for adequate comparison between optimization algorithms. Similar parameters were calculated as specified in [*2*] to quantitatively evaluate the performance of each algorithm, including the probability of success (*P_s_*), *SP1* value, as well as the average and standard deviation of successful runs.

**1.3 Results of the Formal Benchmarking for Single Objective Algorithms**

The results of the benchmark comparison indicated that the AMALGAM-SO optimization algorithm was, on average, superior to the saDE, LSHADE-cnEpSin, and DE algorithms for locating the global minimum for most test functions, which agrees with what was originally expected (Figure S1). This performance was especially apparent for the unimodal test functions, where AMALGAM-SO could quickly and efficiently locate the global minimum values (especially for function # 3, Figure S1). However, as indicated by the high spread of the standard deviation values, it was clear that the performance was quite variable for each algorithm, due to the stochastic nature of each search algorithm. All the algorithms had difficulty solving the multimodal and expanded test functions 8, 13, and 14, which is similar to results presented for other evolutionary optimization algorithms [*2, 5*]. The self-adaptive variant of DE, which switches between mutation strategies based on a stored memory of performance outperformed the AMALGAM-SO algorithm for test function 9 (shifted Rastrigin’s function) as well as test function 15 (the 1^st^ hybrid composition function) (Figure S1). However, the performance of saDE was relatively hindered on the rotated benchmark functions (3, 10, 11), where AMALGAM-SO generally performed well (Figure S1).

The LSHADE-cnEpSin global, single objective optimization algorithm, which relies on a more complex approach to adapt the DE control parameters than saDE or DE (using an ensemble approach with sinusoidal increasing or decreasing adjustments), could outperform both the saDE and DE variants for a variety of the unimodal test problems (1-5, especially apparent for 3) (Figure S1). This algorithm can effectively balance the exploitation of already achieved “best” solutions along with exploration of non-visited regions of the search space and is better equipped to handle search spaces with correlated parameter values as compared to the saDE and DE versions [*9*]. However, for multimodal functions 6 and 7, saDE performs slightly better than LSHADE (Figure S1). Notably, on average, LSHADE outperforms the AMALGAM-SO algorithm for test functions 9, 13, and 15 (Figure S1).


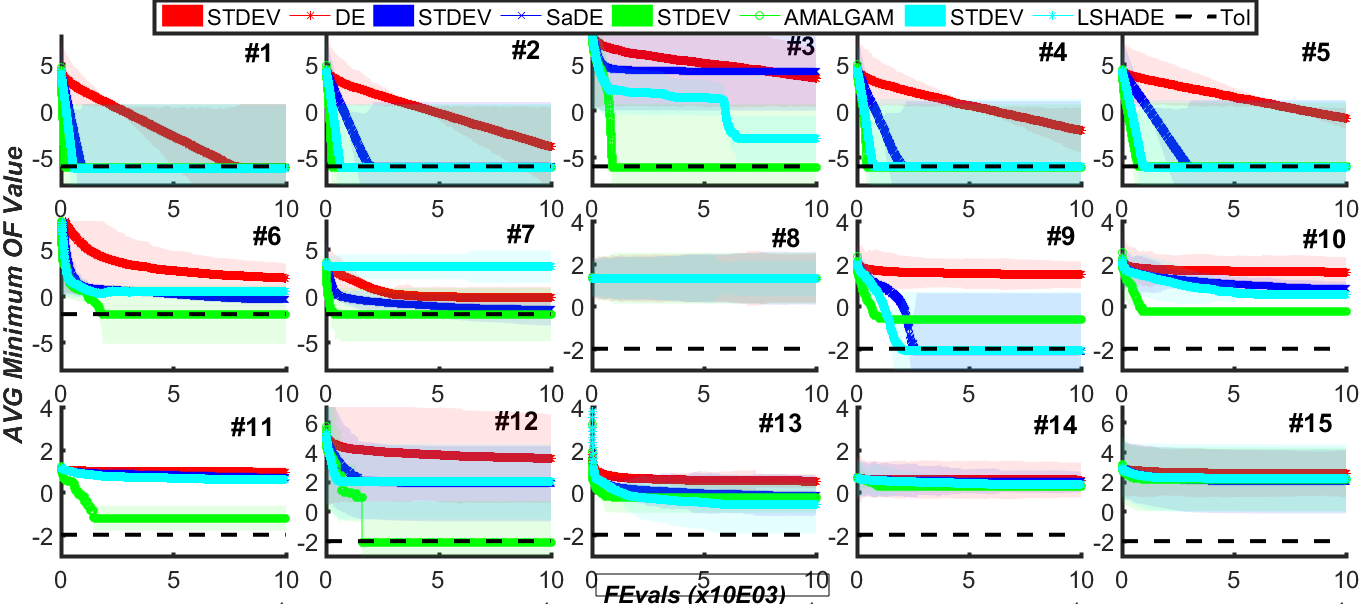


*Figure S1. Comparison of DE, saDE, AMALGAM-SO, and LSHADE-cnEpSin optimization algorithms tested on 15 benchmark functions*

The calculated values of optimization performance were in general agreement with trends illustrated in Figure S1, as demonstrated in Tables S1, S2, S3, and S4. As expected, *SP1* values were generally lower for unimodal as compared to more complex multimodal test functions. The classical DE optimization algorithm was only able to solve test function #1 in the allotted number of function evaluations. All optimization algorithms were unable to solve (at least once) test functions 8, 13, and 14 (with SP1 values and function evaluations approaching 4000 and 100,000 respectively). These results were similar to those observed by [*11*], where no combinations of algorithms of AMALGAM-SO were able to solve these rather complex arrays of hybrid composition test functions. The AMALGAM-SO algorithm used in this benchmark approach performed differently than what was reported in [*6*]. For example, AMALGAM-SO (with CMAES-PSO-GA) did not perform as well on test functions 9, 10, 11, and 15. The reason for these inconsistencies is relatively unknown.

AMALGAM-SO was able to solve the remaining test functions (1-7, 9-12, 15) with a relatively higher probability (*P_s_*) than both the saDE and LSHADE-cnEpSin algorithms (Tables S2 and S3). Exceptions to this trend included test functions 9 and 15, which was similar to what was depicted qualitatively in Figure S1. In addition, AMALGAM-SO was reflected by lower standard deviation values over LSHADE-cnEpSin or saDE, suggesting that the search performance was more reproducible and less stochastic in nature (Tables S2 and S3). Compared to saDE, AMALGAM-SO generally took a much smaller number of function evaluations to reach the global minimum, most likely due to the higher search efficiency afforded by the combination of three premier evolutionary search algorithms. Finally, the average minimum objective function values (MinAVG) and associated standard deviations were generally smaller for the AMALGAM-SO algorithm over saDE or LSHADE-cnEpSin (exceptions for problems 9 and 15). After comparing trends in tabulated Ps and MinSTDEV values, the LSHADE algorithm was slightly more variable in performance than the saDE algorithms, due in part to the adaptation procedure in LSAHDE-cnEpSin (Tables S2 and S4). Overall, AMALGAM-SO demonstrated a more reliable optimization performance than the LSHADE-cnEpSin, saDE and classical DE alone, where the following classification can be concluded: AMALGAM-SO>LSHADE-cnEpSin>saDE>DE.

*Table S1 – Tabulated results of classical DE on 15 benchmark test functions*

| **Test Function** | **Ps** | **SP1** | **AVG** | **STDEV** | **MinAVG** | **MinSTDEV** |
| --- | --- | --- | --- | --- | --- | --- |
| 1 | 1 | 3048 | 76192 | 6577 | 8.1046E-07 | 1.58211E-07 |
| 2 | 0 | 4000 | - | - | 1.23E-04 | 5.54096E-05 |
| 3 | 0 | 4000 | - | - | 2966 | 1195 |
| 4 | 0 | 4000 | - | - | 7.09E-03 | 3.86E-03 |
| 5 | 0 | 4000 | - | - | 1.43E-01 | 7.98E-02 |
| 6 | 0 | 4000 | - | - | 74 | 35.25 |
| 7 | 0 | 4000 | - | - | 6.37E-01 | 7.46E-02 |
| 8 | 0 | 4000 | - | - | 20 | 7.03E-02 |
| 9 | 0 | 4000 | - | - | 30 | 4.29 |
| 10 | 0 | 4000 | - | - | 39.54 | 5.05 |
| 11 | 0 | 4000 | - | - | 8.74 | 0.56 |
| 12 | 0 | 4000 | - | - | 3502 | 921 |
| 13 | 0 | 4000 | - | - | 3.26 | 4.70E-01 |
| 14 | 0 | 4000 | - | - | 3.72 | 1.50E-01 |
| 15 | 0 | 4000 | - | - | 326 | 36 |

*Table S2 – Tabulated results of saDE on 15 benchmark test functions*

| **Test Function** | **Ps** | **SP1** | **AVG** | **STDEV** | **Tolerance** | **MinAVG** | **MinSTDEV** |
| --- | --- | --- | --- | --- | --- | --- | --- |
| 1 | 1 | 362 | 9042 | 296 | 1E-06 | 8.25E-07 | 1.72E-07 |
| 2 | 1 | 712 | 17788 | 1657 | 1E-06 | 8.66E-07 | 1.05E-07 |
| 3 | 0 | 4000 | - | - | 1E-06 | 14019 | 14316 |
| 4 | 1 | 708 | 17704 | 1993 | 1E-06 | 8.78E-07 | 8.706E-08 |
| 5 | 1 | 1166 | 29146 | 870 | 1E-06 | 9.16E-07 | 7.39E-08 |
| 6 | 1 | 3002 | 75042 | 15524 | 1E-06 | 3.32E-01 | 1.10 |
| 7 | 0.52 | 3180 | 60581 | 22162 | 1E-02 | 2.94E-02 | 1.95E-02 |
| 8 | 0 | 4000 | - | - | 1E-02 | 20 | 5.90E-02 |
| 9 | 1 | 908 | 22712 | 1550 | 1E-02 | 8.48E-03 | 1.04E-03 |
| 10 | 0 | 4000 | - | - | 1E-02 | 6.44 | 2.20 |
| 11 | 0.04 | 3998 | 98900 | - | 1E-02 | 4.78 | 1.36 |
| 12 | 0.68 | 2128 | 31176 | 17704 | 1E-02 | 71 | 338 |
| 13 | 0 | 4000 | - | - | 1E-02 | 6.75E-01 | 1.24E-01 |
| 14 | 0 | 4000 | - | - | 1E-02 | 3.03 | 3.24E-01 |
| 15 | 0.36 | 2968 | 35644 | 9907 | 1E-02 | 101 | 141 |

*Table S3 – Tabulated results of AMALGAM-SO on 15 benchmark test functions*

| **Test Function** | **Ps** | **SP1** | **AVG** | **STDEV** | **Tolerance** | **MinAVG** | **MinSTDEV** |
| --- | --- | --- | --- | --- | --- | --- | --- |
| 1 | 1 | 69 | 1737 | 97 | 1E-06 | 7.82E-07 | 1.57E-07 |
| 2 | 1 | 108 | 2688 | 132 | 1E-06 | 7.78E-07 | 1.87E-07 |
| 3 | 1 | 326 | 8152 | 504 | 1E-06 | 7.92E-07 | 1.73E-07 |
| 4 | 1 | 126 | 3160 | 376 | 1E-06 | 7.59E-07 | 1.67E-07 |
| 5 | 1 | 280 | 6997 | 329 | 1E-06 | 8.71E-07 | 1.32E-07 |
| 6 | 1 | 423 | 10568 | 9319 | 1E-06 | 8.98E-03 | 7.40E-04 |
| 7 | 1 | 316 | 7908 | 6913 | 1E-02 | 8.54E-03 | 1.38E-03 |
| 8 | 0 | 4000 | - | - | 1E-02 | 20.368 | 0.064 |
| 9 | 0.72 | 2348 | 42628 | 18906 | 1E-02 | 2.25E-01 | 6.50E-01 |
| 10 | 0.52 | 3118 | 57615 | 18282 | 1E-02 | 5.61E-01 | 6.44E-01 |
| 11 | 0.96 | 1264 | 28745 | 17857 | 1E-02 | 6.04E-02 | 2.56E-01 |
| 12 | 1 | 894 | 22348 | 18753 | 1E-02 | 7.71E-03 | 1.63E-03 |
| 13 | 0 | 4000 | - | - | 1E-02 | 5.41E-01 | 1.99E-01 |
| 14 | 0 | 4000 | - | - | 1E-02 | 1.793 | 0.613 |
| 15 | 0.08 | 3935 | 79420 | 15061 | 1E-02 | 143.200 | 136.926 |

*Table S4 – Tabulated results of LSHADE-cnEpSin on 15 benchmark test functions*

| **Test Function** | **Ps** | **SP1** | **AVG** | **STDEV** | **Tolerance** | **MinAVG** | **MinSTDEV** |
| --- | --- | --- | --- | --- | --- | --- | --- |
| 1 | 1 | 223 | 5568 | 59 | 1E-06 | 6.61E-07 | 1.9E-07 |
| 2 | 1 | 265 | 6634 | 0 | 1E-06 | 6.89E-07 | 1.38E-07 |
| 3 | 0.8 | 3027 | 69608 | 32699 | 1E-06 | 3.95E-05 | 7.85E-05 |
| 4 | 1 | 293 | 7313 | 72 | 1E-06 | 9.38E-07 | 5.4E-08 |
| 5 | 1 | 390 | 9740 | 195 | 1E-06 | 7.54E-07 | 5.94E-08 |
| 6 | 0.32 | 3129 | 31959 | 4839 | 1E-06 | 2.71E+00 | 1.89 |
| 7 | 0.52 | 4000 | 100000 | 0 | 1E-02 | 1.21E+03 | 7.23E+01 |
| 8 | 0 | 4000 | - | - | 1E-02 | 20 | 8.73E-02 |
| 9 | 1 | 644 | 16098 | 1594 | 1E-02 | 8.17E-03 | 1.77E-03 |
| 10 | 0.04 | 3999 | 99530 | - | 1E-02 | 3.58 | 1.60 |
| 11 | 0 | 4000 | - | - | 1E-02 | 3.81 | 1.03 |
| 12 | 0.52 | 2083 | 7838 | 2731 | 1E-02 | 97 | 336 |
| 13 | 0 | 4000 | - | - | 1E-02 | 2.20E-01 | 4.40E-02 |
| 14 | 0 | 4000 | - | - | 1E-02 | 2.24 | 6.05E-01 |
| 15 | 0.44 | 2716 | 27057 | 13519 | 1E-02 | 161 | 189 |

**2.0 Formal Testing of the Model Prediction Residuals**

This section confirms the validity of several assumptions made when deriving the maximum likelihood function used for the GSO and GMO algorithms in this study. The four main assumptions made in this derivation were as follows:

1. The error residuals are normally distributed with zero mean;
2. The error residuals are independent;
3. The error residuals are homoscedastic (constant variance).

Regarding the first assumption, quantile-quantile (QQ) plots of the best performing parameter sets for each study (using the Moser model for Studies 1-3 and Heijnen model for Study 4) indicated that the error residuals for both substrate and cell concentration predictions were normally distributed, as all residuals fell close to the QQ line plot (Figure S2) [*16, 17*]. In addition, an Anderson-Darling (AD), one-sample Komolgorov-Smirnov (KS) test, and D’Agostino Pearson K2 test were performed to quantitatively assess if the error residuals were normally distributed [*17-19*]. Both the AD and KS tests evaluate the significance of the departure of an empirical cumulative distribution function (CDF) created from the data from the CDF of a hypothetical normal distribution (where the KS significance test is nonparametric and the AD test places more emphasis on the tails of the empirical distribution), whereas the K2 test evaluates the normality based on measures of skewness and kurtosis of the data alone [*17-19*].

The results demonstrated that for most Studies (2-4), the null hypothesis that the distribution of error residuals was normal was not rejected, as the calculated p-values were mostly greater than 0.05 (Table S5). Although rejection of the null hypothesis was not observed for the AD test for Study 1, the KS test indicated otherwise (Table S5). Similarly, the null hypothesis was rejected for the cell concentration residuals obtained from Study 2, indicating the distribution of residuals was not normal based on measures of skewness and kurtosis alone (Table S5). We also quantitatively checked if the mean of the residuals for each Study was significantly different from 0 using the F-test statistical approach presented in [*20*]. The confidence level values calculated ranged from 61% to 92%, indicating that there was generally high certainty (greater than 90% for Studies 1,2 and 4) that the mean of the error residuals was 0; however, these results were generally not statistically significant using the 0.05 significance level.

*Table S5 – Results of the normality testing significance (p) values for residuals from predicted substrate and cell concentrations*

| **Study** | **AD Test** | | **KS Test** | | **K2 Test** | |
| --- | --- | --- | --- | --- | --- | --- |
|  | **p-value (Substrate)** | **p-value  (Cells)** | **p-value (Substrate)** | **p-value  (Cells)** | **p-value (Substrate)** | **p-value (Cells)** |
| 1 | 0.297 | 0.149 | 0.0148 | 0.0095 | 0.463 | 0.291 |
| 2 | 0.527 | 0.092 | 0.264 | 0.276 | 0.691 | 0.0378 |
| 3 | 0.869 | 0.257 | 0.228 | 0.131 | 0.858 | 0.158 |
| 4 | 0.581 | 0.749 | 0.236 | 0.141 | 0.694 | 0.564 |


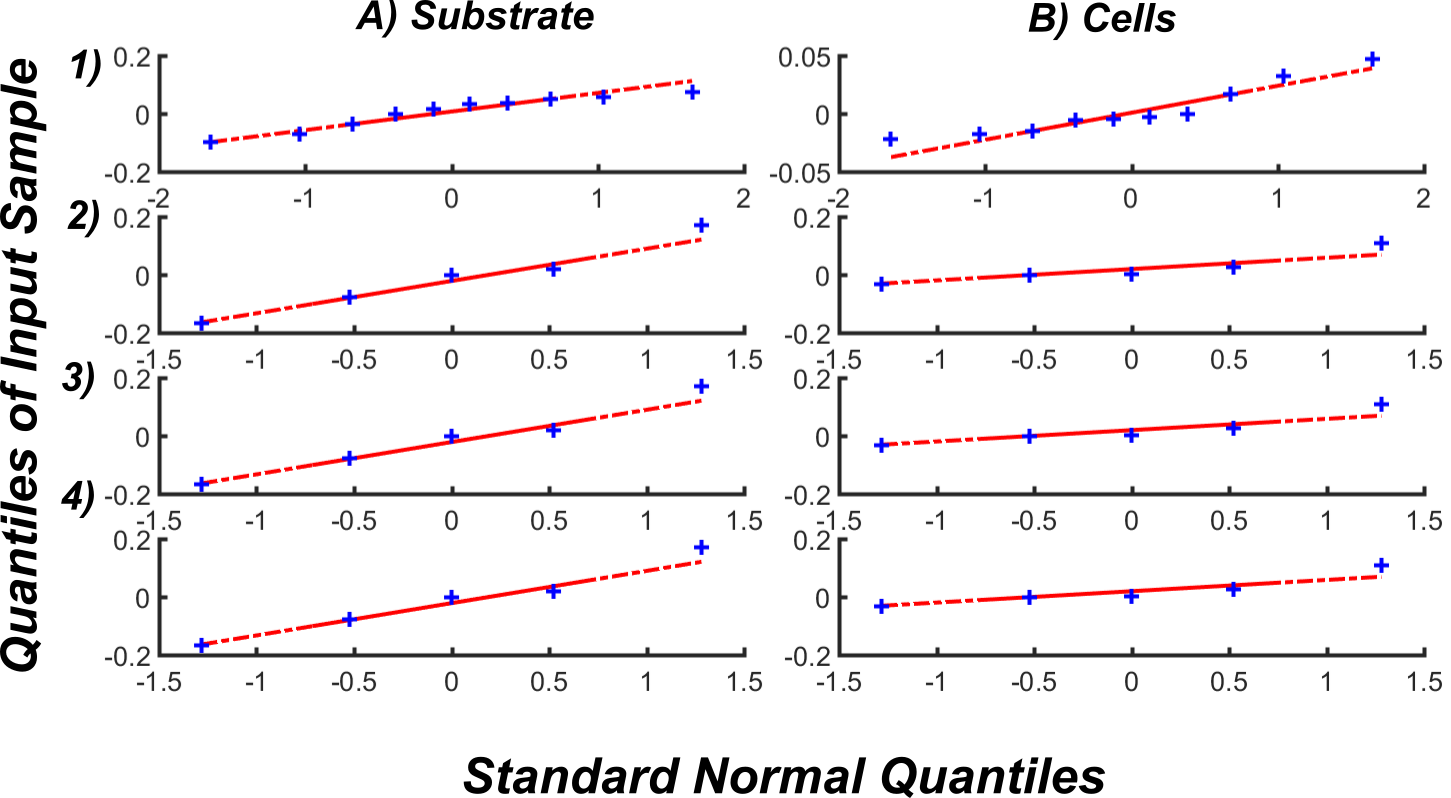

*Figure S2. Quantile-quantile plots for the best fitting parameter sets obtained for studies 1-4, separated by residuals pertaining to A) substrate and B) cell concentration variables.*

To check if the error residuals were independent, several methods were investigated. Autocorrelation plots were first developed to qualitatively inspect whether the error residuals were independent and non-correlated (Figure S3) [*21, 22*]. As observed in Figure S2, most of the autocorrelation values were close to 0, especially for Studies 2-4, which demonstrates that there was little temporal similarity among error residuals. In addition, most of the autocorrelation estimates ranged within the 95% standard error bounds, signifying that the autocorrelation response was relatively uniform across different lag time periods. For some initial lag periods (i.e., 1), the autocorrelation values were high, especially for Study 1; however, the values seemed to dampen as the number of lag periods progressed, which was indicative of independent residual distributions for both predicted substrate and cell concentrations.

The Ljung Box Q, Runs test, and Turning point tests were used to quantitatively assess the independence of the residual errors for each study [*21, 23-25*]. The Ljung Box Q test investigates the null hypothesis that the residuals are not autocorrelated using a chi-squared statistical test [*21*]. This test depends on the number of lags (*L*) incorporated, where we varied the number of lags from 1 to the number of observations and reported the minimum p-value from these estimates. Based on Table S6, a majority of the p-values were greater than 0.05, suggesting that the null hypothesis was not rejected and that the residuals are indeed not autocorrelated. The only exception to these results was the error residuals obtained from the substrate concentration predictions in Study 1, which resulted in a rejection of the null hypothesis.

The Runs test, on the other hand, tests the null hypothesis that the residuals come in random order, and can help support the hypothesis that they are independently distributed in time or space [*23-25*]. For all studies, the p-value was greater than 0.05, signifying that the null hypothesis was not rejected, and that the error residuals were indeed in random, independent ordering (Table S6).

Finally, the Turning test (based on the number of up or down turns) tests the null hypothesis that the set of residuals are independent and identically distributed, random values. If the value of the *Nt* statistic calculated from this test is greater than 1.96, then the null hypothesis is rejected, and the values do not come from an independent, identically distributed set of random numbers [*23-25*]. The results in Table S6 indicated that most of the residuals were indeed independent and identically distributed random numbers, except for the residuals obtained from the substrate predictions in Study 1, as the Nt statistic values were generally below the 1.96 critical threshold.

*Table S6 – Results of the independence testing significance (p) values for residuals from predicted substrate and cell concentrations*

| **Study** | **Ljung Box Q Test** | | **Runs Test** | | **Turning Test** | |
| --- | --- | --- | --- | --- | --- | --- |
|  | **p-value**  **(Substrate)** | **p-value**  **(Cells)** | **p-value**  **(Substrate)** | **p-value**  **(Cells)** | **Nt-value**  **(Substrate)** | **Nt-value**  **(Cells)** |
| 1 | 0.0204 | 0.0931 | 0.095 | 0.167 | 2.98 | 0.916 |
| 2 | 0.166 | 0.485 | 1 | 1 | 1.76 | 0 |
| 3 | 0.0977 | 0.859 | 1 | 1 | 1.76 | 1.76 |
| 4 | 0.139 | 0.899 | 1 | 1 | 0 | 1.76 |


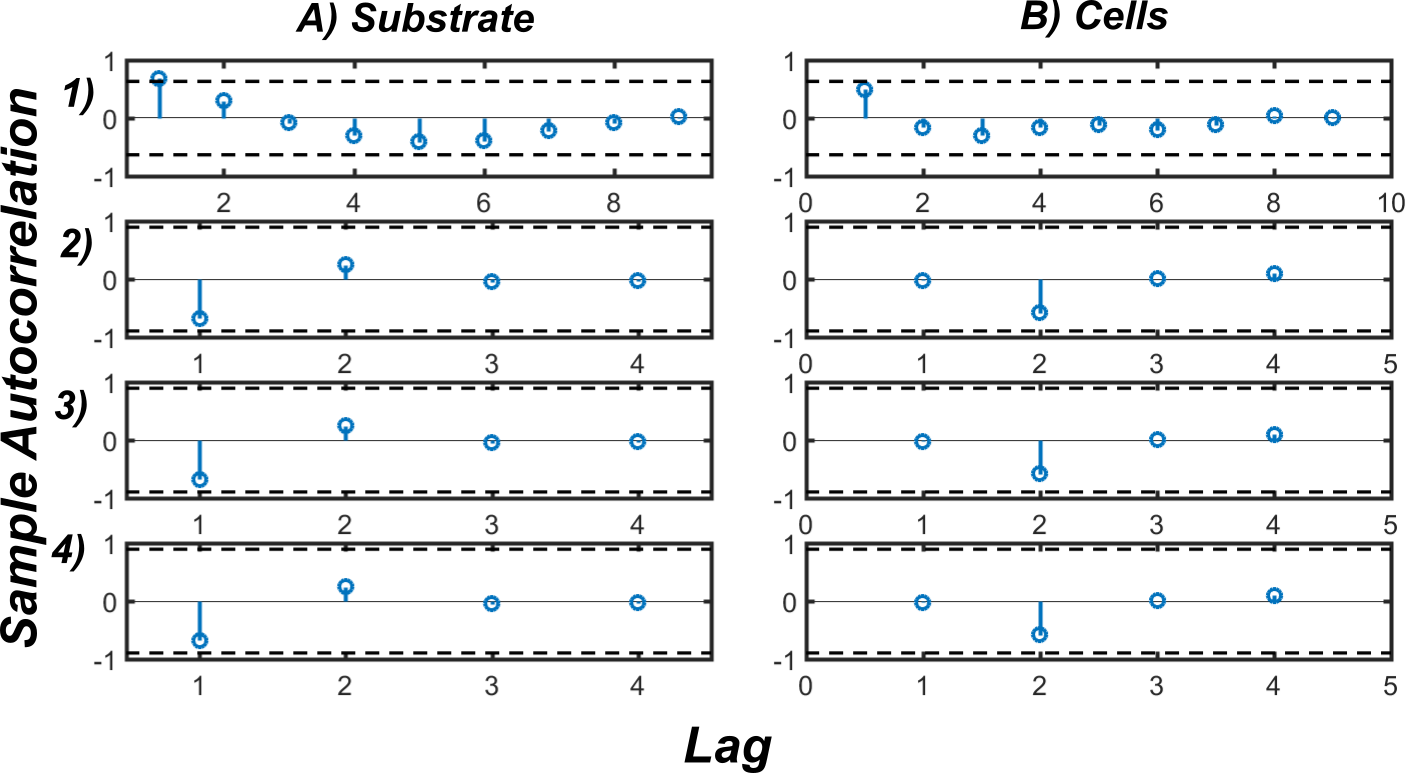


*Figure S3. Autocorrelation plots for the best fitting parameter sets obtained for studies 1-4, separated by residuals pertaining to A) substrate and B) cell concentration variables.*

To check for homoscedasticity, Engle’s ARCH test [*26*], the Bresuch and Pagan [*27*], and the White test [*28, 29*] methods were applied. Engle’s ARCH test tests the null hypothesis that a series of residuals exhibits no conditional heteroscedasticity by fitting an ARCH(*L*) model to the residuals, where *L* represents the number of lags included [*26*]. Similar to the Ljung Box Q Test, we varied the number of lags included to fit/develop the ARCH model from 1 to the number of observations and reported the minimum p-value from these estimates. Based on this test, the null hypothesis was not rejected for any study, as the distribution of p-values never dropped below the 0.05 significance level (Table S7). Thus, we can conclude with high statistical certainty that there was no conditional heteroscedasticity to the residuals obtained from the predictions of substrate or cell concentrations for each study.

The Breusch-Pagan and White methods test whether the variance of the error residuals from a regression are dependent on the values of the independent “predictors,” indicating heteroscedasticity [*26-29*]. Here, the “predictors” are the predicted substrate and cell concentrations from the nonlinear kinetic models. The Breusch-Pagan method differs from the White method as it considers a simple, additive linear regression between the squared residuals and the predictors, whereas the White method considers a more complex quadratic regression between the squared residuals and predictors. Both tests test the null hypothesis that the error residuals are homoscedastic using a chi-squared statistical significance test. Different results were observed between the two methods, as the White test generally did not reject the hypothesis that the error residuals were homoscedastic (as all p-values > 0.05), whereas the Breusch-Pagan test rejected the null hypothesis for the residuals obtained from substrate predictions for Studies 2-4 (Table S7). Clearly, if the datasets were not so sparse, and included higher resolution at more dynamic portions of the kinetic curves, the validity of these statistical tests would be greatly improved. As each of these tests for homoscedasticity relies on some form of regression, clearly the predictive power of the regression models (and strength of the statistical inferences achieved from hypothesis testing) will be greatly improved as the number of datapoints used for calibration increases. Therefore, based on the sparsity of the datasets acquired (and lack of experimental replications), it is difficult to fully justify the assumption of homoscedasticity among the error residuals.

*Table S7 – Results of the heteroscedasticity testing significance (p) values for residuals from predicted substrate and cell concentrations*

| **Study** | **Engle’s ARCH Test** | | **Breusch-Pagan Test** | | **White Test** | |
| --- | --- | --- | --- | --- | --- | --- |
|  | **p-value**  **(Substrate)** | **p-value**  **(Cells)** | **p-value**  **(Substrate)** | **p-value**  **(Cells)** | **p-value**  **(Substrate)** | **p-value**  **(Cells)** |
| 1 | 0.0643 | 0.255 | 0.132 | 0.551 | 0.246 | 0.475 |
| 2 | 0.183 | 0.223 | 0.0275 | 0.667 | 0.0827 | 0.098 |
| 3 | 0.165 | 0.223 | 0.0278 | 0.552 | 0.088 | 0.566 |
| 4 | 0.0981 | 0.223 | 0.0284 | 0.271 | 0.087 | 0.532 |

**3.0 Formal Comparison of Multi Objective Optimization Algorithms**

A total of 10 evolutionary, multi-objective optimization algorithms (EMOA) were formally benchmarked against 13 test functions. The EMOA algorithms selected for screening in this study were considered from the results presented in Tanabe et al. [*30*] (for M = 2 objective functions, similar conditions to our problem herein), which benchmarked 21 recent and classical EMOA algorithms using the WFG suite of test functions. The 10 selected algorithms included: MO-CMA-ES (the multi-objective version of CMA-ES [*31*]; NSGA-III [*32*]; MOEA/D [*33*]; MOEA/DD [*34*]; SPEA2-SDEA [*35*]; IBEA [*36*]; RVEA [*37*]; MOEA/IGDNS [*38*]; AMALGAM-MO [*2*]; and A-NSGA-III [*39*]. For the MO benchmark tests, we selected test functions that were both non-convex (WFG 1-9) and convex (MaF3, MaF5, MaF11, MaF15), multi-modal, biased, separable, and non-separable to gain a full perspective of the performance of each algorithm. Importantly, the ratio of convex to concave problems was set at 1:1 to ensure that performance evaluations were equally split between these two specific categories of test problems (even though the MO problems in this study were convex). Each of these test algorithms and problems (except AMALGAM-MO) were compiled by Tian et al. [*40*] in a convenient platform for testing and comparison (PlatEMO v.3.0), which was utilized in this study.

The metrics used to compare the EMOA algorithms differed significantly from the SO algorithms, as it is impossible to judge the performance of an EMOA using one singular criterium. Similar to criteria reviewed by [*41*], the EMOA algorithms were compared using the final, non-dominated Pareto solution sets returned by each optimization run (which was different than criteria presented by [*30*]). The algorithms were judged based on three categories: accuracy and convergence (how close the solution sets were to the exact Pareto front), diversity (distribution of solutions and spread/coverage), and the number of nondominated solutions in the experimental solution set [*41*]. Accuracy and convergence were evaluated based on five primary parameters: set coverage (C, higher number is better), final generational distance (GD, lower number), inverted generational distance (IGD, lower number), hypervolume (HV, higher number), and normalized hypervolume (NHV, higher number), described in detail elsewhere [*42-46*]. It is important to note that C, IGD. HV, and NHV metrics also consider diversity/uniformity of solution sets in addition to accuracy and convergence. Diversity or uniformity of solution sets were judged using the following parameters: spacing (S), spread (Sp), and pure diversity (PD). The number of nondominated solutions in the experimental Pareto front was assessed using the RNI metric (ratio of nondominated solution sets) [*45*]. In addition, the CPU time was compared among algorithms, yet another factor to benchmark for each test function.

To fairly assess the performance of each algorithm over the wide range in test problems, a scoring system was constructed for each metric (excluding coverage and RNI statistics). In this approach, a score on the scale of 1-10 (10 being ‘excellent’ and 1 being ‘poor’) was delegated to each algorithm after sorting the calculated results for each metric (in either ascending or descending order, depending on the metric chosen). For this scoring method, a perfect performance would be equivalent to a score of 1040 (first place for each problem and metric), whereas a very poor performance would be equivalent to 0. The coverage and RNI scores were excluded from this comparison as each algorithm reported many similar values for each of these metrics, making it difficult to find a rank for comparison.
 **3.1 Run Conditions and Control Settings for Multi-Objective Algorithms**

The AMALGAM-MO run conditions and control settings used in this study are summarized in Table S8. Run conditions were kept identical to those developed for the other two MO algorithms (fixed population and generations). All the control settings required to run this algorithm were identical to the default values presented in [*47*], including all the recombination methods listed. Latin hypercube sampling was used for sampling from the initial prior distribution. Boundary handling was set to the reflect option, where mutated values outside the feasible range were reflected an equal distance back into the feasible parameter space. DE scaling factors (i.e., *F_de_*) and PSO inertia factors were set to uniform distributions (U) using the ranges specified in Table S8.

*Table S8 – Run conditions and control settings for the AMALGAM-MO application*

| **Parameter Setting** | **Nominal Value** |
| --- | --- |
| Number of Parameters (D) | 6 |
| Population Size (N) | 100 |
| Number of Generations (T) | 20,000 |
| Number of Objective Functions (m) | 2 |
| Prior Distribution | Latin |
| Boundary Handling | Reflect |
| Recombination Methods | GA, PSO, AMS, DE |
| NSGA Crossover Probability | 0.9 |
| NSGA Mutation Probability | 1/6 |
| NSGA Cross Distribution Index | 10 |
| NSGA Mutation Distribution Index | 50 |
| AMS Jump Rate | 0.9440 |
| DE Scaling Factor – DE Variant 1 | U[0.6,1] |
| DE Scaling Factor – DE Variant 2 | U[0.2,0.6] |
| PSO Social Factor | 1.5 |
| PSO Cognitive Factor | 1.5 |
| PSO Inertia Factor | U[0.5,1] |
| Thinning Rate | 1 |
| Minimum Selection Probability | 0.05 |

EMOA run conditions were fixed for each test problem to settings that resembled the unique problems presented in this study including M=2 objective functions, d = 6 “parameters”, N = 100 population members, and 50*10^4^ function evaluations. The SBX crossover and polynomial mutation functions were selected for each run, as suggested by [*30*] and provided by the PlatEMO user interface. The user settings for the crossover and mutation functions were identical to those presented in Tanabe et al. 2017 (pc = 1, nc = 30, pm = 1/d, and nm = 20). Importantly, each algorithm was run for five individual replicates and an average metric was computed and recorded for each test problem to gain some statistical significance in the comparison evaluation.

The results of the formal comparison demonstrated that the NSGA-III algorithm was the most robust multi objective optimizer to the wide range in test problems encountered, whereas the adaptive NSGA-III algorithm performed the worst (Table S9). Interestingly, although AMALGAM-MO combines several different algorithms (i.e., PSO, NSGA-II, DE) to improve MO performance, it was still outperformed by the NSGA-III algorithm. This result was unexpected; however, the relatively short run time (50,000 function evaluations) may have hindered the performance of the AMALGAM-MO algorithm. Future comparisons should be made between all algorithms at a higher number of function evaluations to clarify this point. The recently introduced MOEA/IGD-NS algorithm also showed a good overall performance across the 13 benchmark test functions (Table S9).

In addition, the percent contribution of each metric to the total score is summarized in Table S9. For the top two performing algorithms, the contribution from each metric to the total score was relatively equivalent (ranging from 9-14%). NSGA-III was slightly more accurate (with higher contributions from HV/NHV metrics) as compared to AMALGAM-MO. However, the distribution of solutions along the pareto front were perhaps more uniform (higher Spacing contribution) and diverse (higher PD contribution) for AMALGAM-MO over NSGAIII. The MOEA/IGD-NS algorithm suffered from a high CPU-time and accuracy (low CPU/GD contribution) but gained a competitive advantage in the distribution and diversity criteria (PD, Spacing, Spread) of the experimental solution sets generated. The remaining algorithms demonstrated a wider spread in the contribution from each metric to the total score as compared to the top three performing algorithms (from 6-37%), where higher contributions came from CPU time or diversity/distribution metrics as opposed to accuracy metrics.

*Table S9 – Comparison of the MO algorithms performance against 13 benchmark test functions*

| **Rank** | **Algorithm** | **Total Score** | **CPU Time (%)** | **GD (%)** | **IGD (%)** | **Spacing (%)** | **Spread (%)** | **HV (%)** | **NHV (%)** | **PD**  **(%)** |
| --- | --- | --- | --- | --- | --- | --- | --- | --- | --- | --- |
| 1 | NSGA-III | 774 | 13 | 13 | 14 | 11 | 11 | 14 | 14 | 9 |
| 2 | AMALGAM-MO | 747 | 12 | 10 | 14 | 14 | 11 | 13 | 13 | 14 |
| 3 | MOEA/IGDNS | 673 | 4 | 11 | 14 | 16 | 18 | 11 | 11 | 14 |
| 4 | RVEA | 643 | 20 | 8 | 12 | 11 | 15 | 11 | 11 | 13 |
| 5 | SPEA2-SDE | 574 | 7 | 15 | 13 | 11 | 10 | 17 | 17 | 11 |
| 6 | MOEA-DD | 568 | 3 | 16 | 17 | 10 | 14 | 16 | 16 | 9 |
| 7 | IBEA | 536 | 14 | 16 | 9 | 10 | 7 | 14 | 14 | 17 |
| 8 | MOEA-D | 470 | 10 | 11 | 11 | 17 | 19 | 10 | 10 | 10 |
| 9 | MO-CMA | 421 | 16 | 9 | 11 | 12 | 13 | 10 | 10 | 20 |
| 10 | A-NSGAIII | 314 | 37 | 18 | 6 | 15 | 5 | 6 | 6 | 6 |

Visual inspection of the performance of the best performing algorithm (NSGA-III) qualitatively confirms the high accuracy and precision of the NSGA-III MO algorithm (Figure S4). Average results from each independent run (from N=5 repetitions) showed that the experimental, non-dominated solutions from the NSGA-III algorithm well approximate the true Pareto front for many of the test functions, both concave and convex in nature. The largest error between the true Pareto front and the experimental front was observed for the WFG-8 test function, which was similar to the performance among all other algorithms. In addition, the coverage of solutions for the WFG1 and 2 as well as MaF11 staircase functions is relatively sparse at lower values of objective function 1 (higher values of objective function 2), where the lack of diversity in NSGA-III solutions for some test problems was similarly confirmed in Table 2 above (Figure S4). These results highlight NSGA-III as a reliable and consistent multi-objective optimization algorithm that will be used as a primary algorithm for model-data calibration in this study.


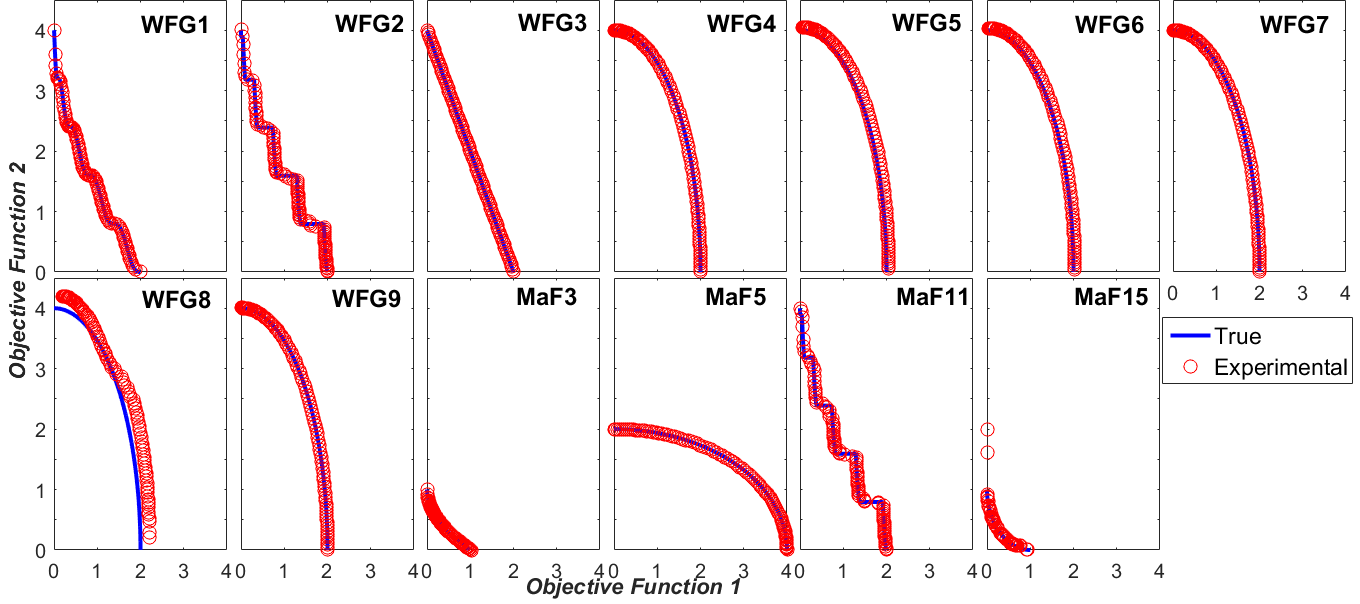


*Figure S4. NSGA-III MO performance when benchmarked against 13 test functions (for M=2 objective functions). The blue line indicates the true Pareto front, whereas the red circles indicate the experimental Pareto front as determined using the NSGAIII algorithm.*

**4.0 DREAM-ZS Run Conditions: Approximate Bayesian Computation**

The DREAM-ZS run conditions for the Approximate Bayesian Computation algorithm are summarized in Table S10. To run the ABC component within DREAM-ZS, the ABC objective function had to be explicitly specified in the DREAM-ZS calling script. Of the two functions provided, the distance function was chosen with a threshold ($\varepsilon_{j}$) value set to 0.025 as recommended in [*48*]. Depending on the model structure and the experimental dataset applied, the number of generations was varied from 50,000 to 400,000 until convergence was formally reached (using the Gelman-Rubin convergence statistic threshold [*49*]). The prior distribution of model parameters was always set to a uniform distribution within the specified realistic uncertainty range. The remaining parameter values specified in Table 2 were found to be optimal to improve convergence speed and were primarily based on information presented in [*50*] and by trial and error approaches.

*Table S10 – DREAM-ZS Parameter Settings for the ABC Analysis*

| **Parameter Setting** | **Nominal Value** |
| --- | --- |
| Number of Parameters (D) | 6 |
| Objective Function | ABC Distance Function (Option 22) |
| Number of Markov Chains (N) | 6 |
| Number of Generations (T) | 50,000-400,000 |
| Prior Distribution | Uniform |
| Boundary Handling | Reflect |
| Number of Crossover Values (nCR) | 3 |
| Number of Chain Pairs for Proposal (delta) | 3 |
| Random Error for Ergodicity (lambda) | 0.1 |
| Randomization (zeta) | 10E-12 |
| Probability of Jump Rate | 0.2 |
| Adapt Selection Probability Crossover | Yes |
| Scaling Factor of Jump Rate (b0) | 1 |
| Epsilon Value | 0.025 |

**5.0 Constrained Boundaries for Improved *FMINCON* Search**Lower and upper parameter boundaries for the improved FMINCON local search are presented in Table S11 for reference. The limits on each parameter were set very close to the optimal parameter sets determined by the global optimization algorithms to gauge the optimization performance of the local search methods around the global optimum.

*Table S11 – Summary of constrained lower (top row) and upper (bottom row) parameter boundaries for the improved FMINCON optimization tests*

| **Study** | **µ_max_** | **K_s_** | **Y** | **K_d_** | **b** | **n** |
| --- | --- | --- | --- | --- | --- | --- |
| 1 | 1.06 | 219 | 2.60 | 0.12 | 0.01 | 2.17 |
|  | 1.08 | 220 | 2.80 | 0.13 | 0.011 | 2.19 |
| 2 | 6 | 0.07 | 3 | 0.8 | 0.07 | 9 |
|  | 7 | 0.09 | 4 | 0.9 | 0.08 | 10 |
| 3 | 1.2 | 0.0001 | 1.7 | 0.01 | 0.000000000075 | 2.2 |
|  | 1.3 | 0.0002 | 1.8 | 0.02 | 0.00000000008 | 2.3 |
| 4 | 2.7 | 0.0001 | 3.3 | 0.02 | 0.6 | 2.6 |
|  | 2.8 | 0.0002 | 3.4 | 0.03 | 0.7 | 2.7 |

**6.0 Formal Convergence of DREAM-ABC vs. DREAM-GL (Gaussian Likelihood) Bayesian Optimization Approaches**

In this section, the formal convergence efficiency of the Approximate Bayesian Computational (ABC) variant of the Bayesian optimization approach DREAM was compared to that of the standard DREAM variant incorporating a formal Gaussian Likelihood objective function. The results are specifically presented for the Moser model calibration. Both the mean and standard deviation from five independent realizations of the overall R statistic of [49] were used to compare the convergence efficiency and reliability for both approaches. For all studies (a-d), the DREAM-GL (Gaussian Likelihood) approach demonstrated improved formal convergence efficiency over the DREAM-ABC approach (Figure S5). Both approaches formally converged by at least 50,000 generations, except for Study 3, which clearly required a larger number of generations before convergence was feasible using both approaches (Figure S5). Apart from Study 3 results, the DREAM-ABC approach was marked by a more consistent optimization performance as compared to the DREAM-GL approach, as reflected by the small standard deviation in the R-statistic across the repetitions performed (Figure S5).


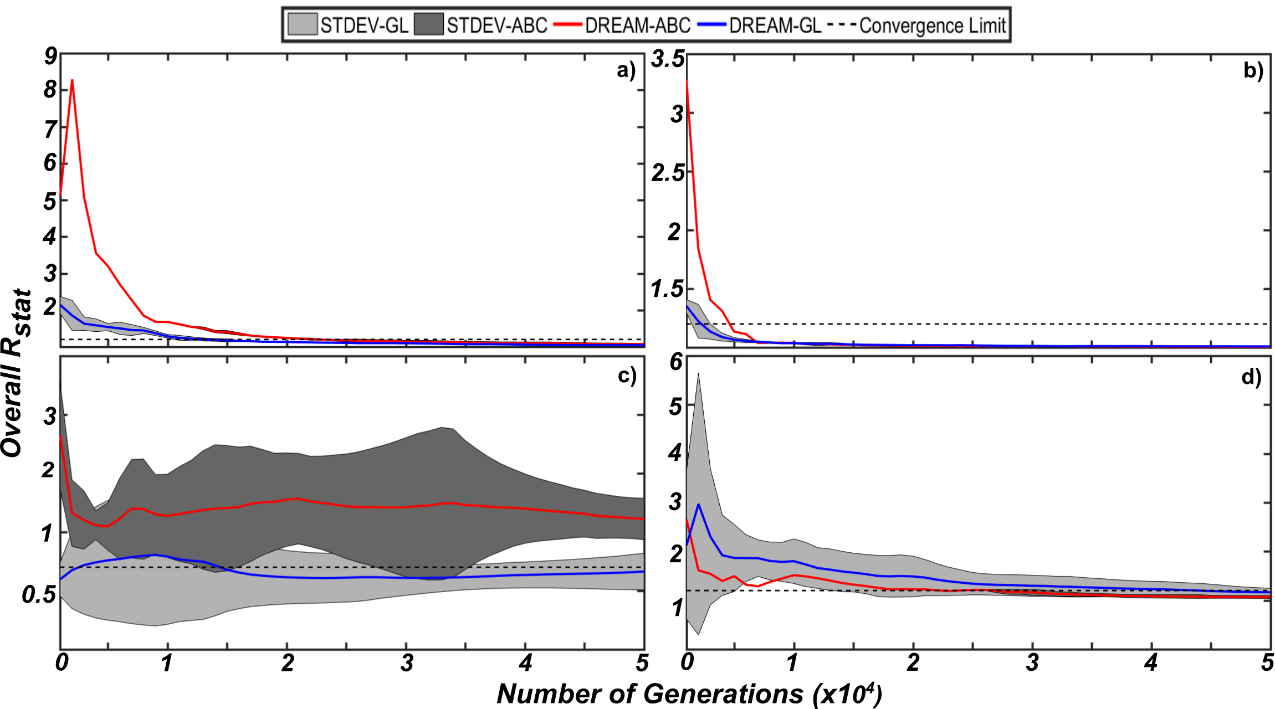

*Figure S5. Evolution of the overall Gelman and Rubin R-statistic for both the DREAM-ZS (ABC) algorithm (red) and the DREAM-ZS (Gaussian Likelihood) algorithm when calibrated against the Moser model for each corresponding dataset (1-4). The letters a-d correspond to Studies 1-4. The solid lines and grey shaded areas indicate the mean value and one standard deviation from the mean value across five independent realizations. The dashed line indicates the convergence threshold of 1.2.*

**7.0 Parameter Identifiability of DREAM-ABC vs. DREAM-GL (Gaussian Likelihood) Bayesian Optimization Approaches**Figures S6-S10 summarize the experimental results comparing the parameter identifiability between the DREAM-ABC and DREAM-GL approaches for Studies 1-3, respectively. For all studies, the parameter identifiability and definition were drastically improved using the DREAM-ABC approach. It is apparent that the Bayesian optimization has not converged for Study 3, as the mean parameter values from the ABC approach were not homogenous, especially for *µ_max_*, *K_s_*, and *Y* model parameters. In addition, some parameters were not identifiable, even when the DREAM-ABC approach was used, as observed for most *Y* values and some *K_d_* values across all studies (Figures S6-S10).


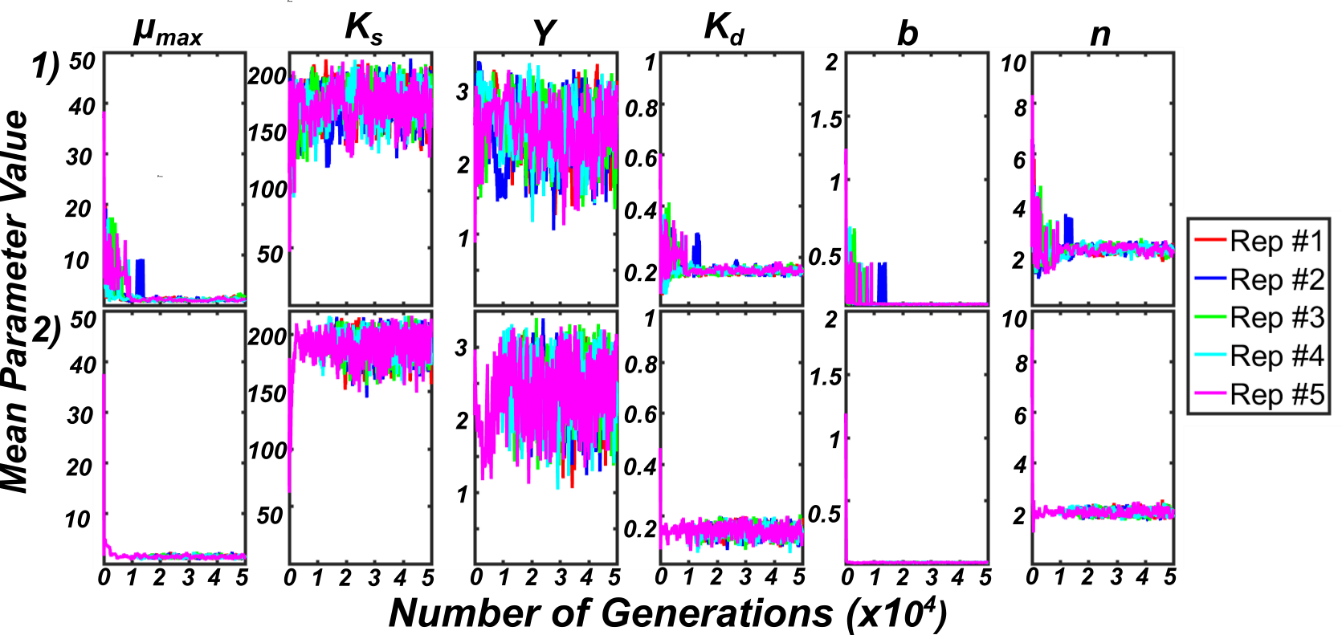

*Figure S6 – Evolution of the mean (across all Markov chains) parameter values for both the 1) DREAM-ZS (Gaussian Likelihood) and 2) the DREAM-ZS (ABC) algorithms when calibrated against the Moser model using the first experimental dataset. The results of five independent repetitions are presented, as differentiated by the color scale of the legend.*


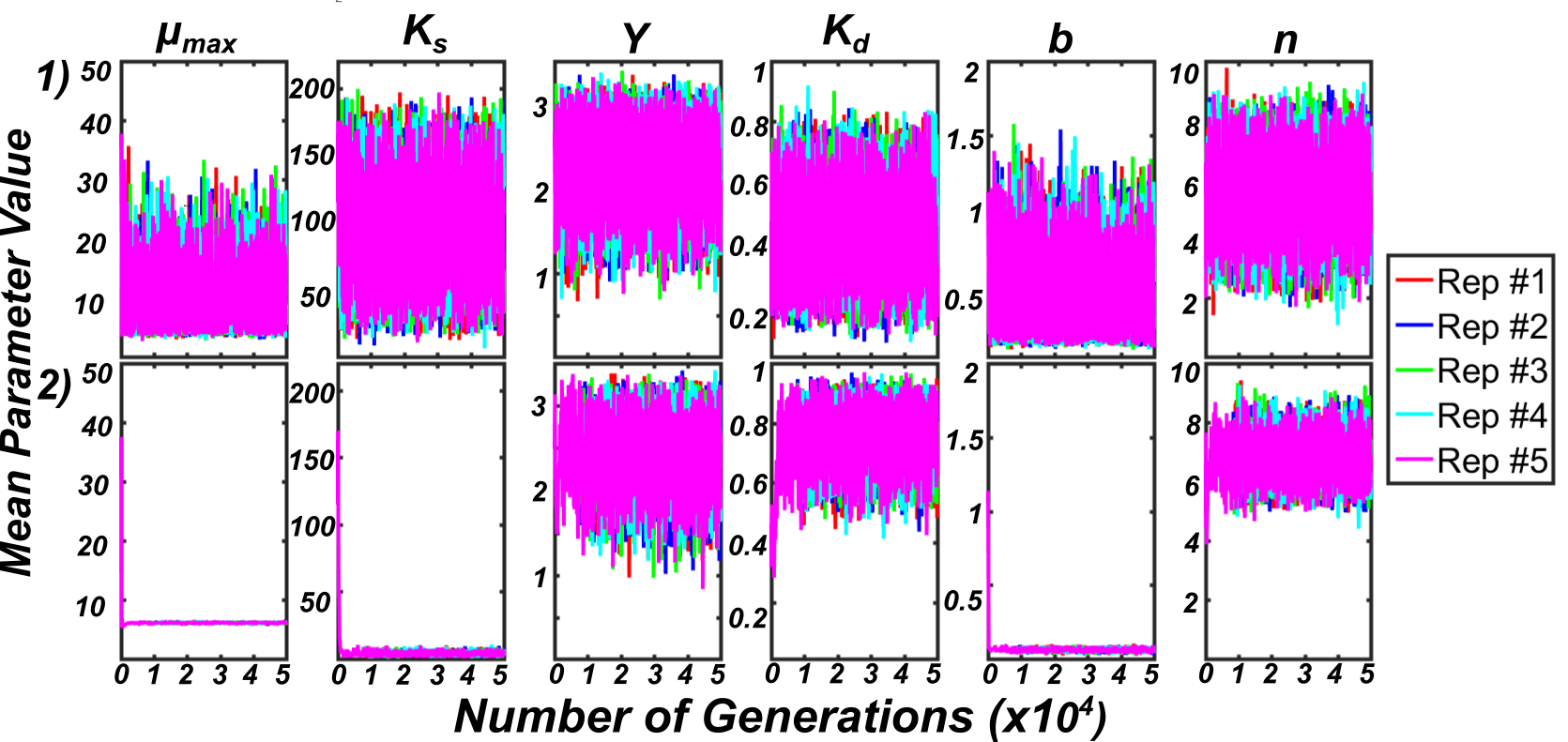

*Figure S7 – Evolution of the mean (across all Markov chains) parameter values for both the 1) DREAM-ZS (Gaussian Likelihood) and 2) the DREAM-ZS (ABC) algorithms when calibrated against the Moser model using the second experimental dataset. The results of five independent repetitions are presented, as differentiated by the color scale of the legend.*


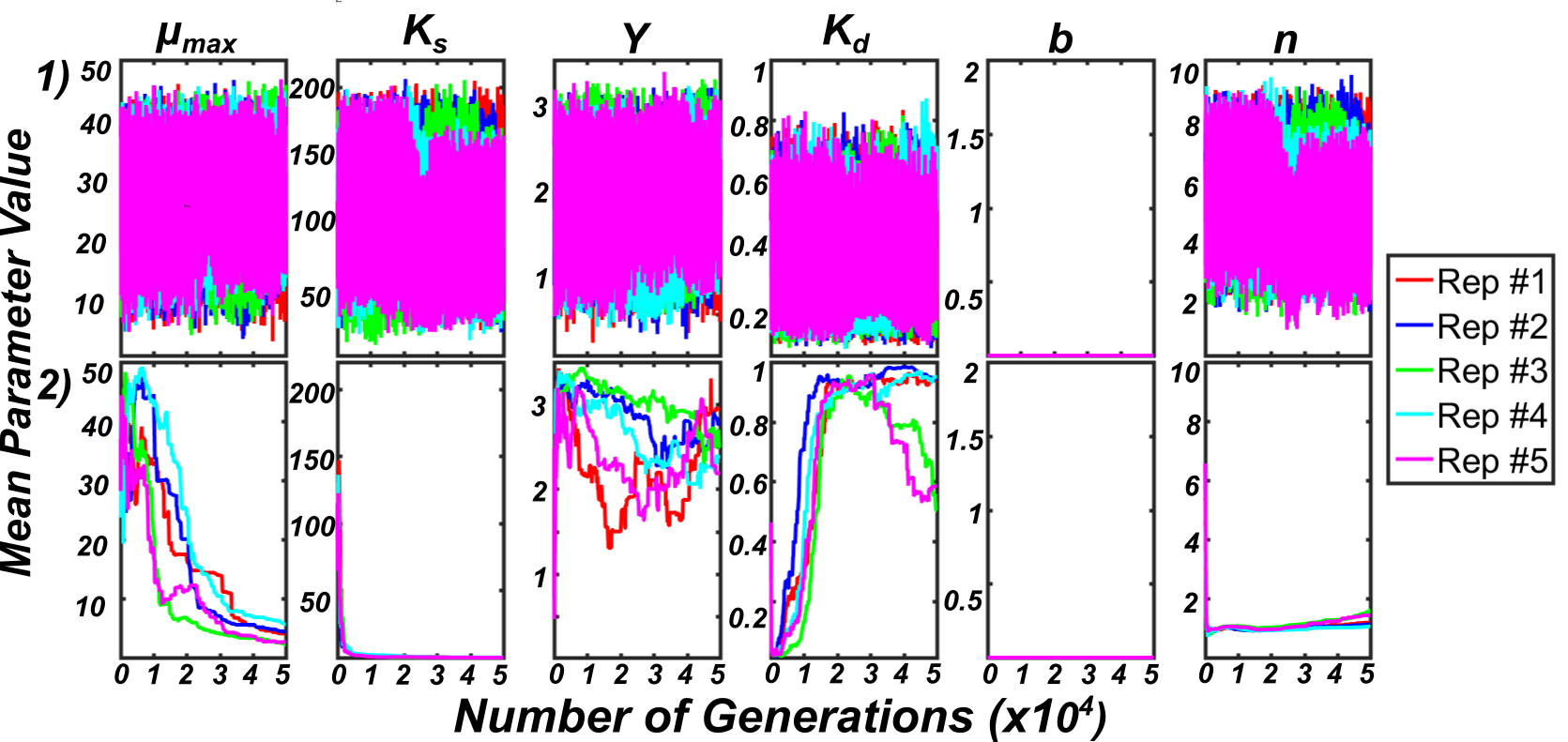

*Figure S8 – Evolution of the mean (across all Markov chains) parameter values for both the 1) DREAM-ZS (Gaussian Likelihood) and 2) the DREAM-ZS (ABC) algorithms when calibrated against the Moser model using the third experimental dataset. The results of five independent repetitions are presented, as differentiated by the color scale of the legend.*

**References**

1. Suganthan, P.N., Hansen, N., Liang, J.J., Deb, K., Chen, Y.P., Auger, A., Tiwari, S., 2005. Problem Definitions and Evaluation Criteria for the CEC 2005 Special Session on Real-Parameter Optimization (KanGAL Report No. 2005005). Kanpur Genetic Algorithms Laboratory, IIT Kanpur).
2. Vrugt, J.A., Robinson, B.A., 2007. Improved evolutionary optimization from genetically adaptive multimethod search. PNAS 104, 708–711.
3. Storn, R., Price, K., 1997. Differential Evolution – A Simple and Efficient Heuristic for global Optimization over Continuous Spaces. Journal of Global Optimization 11, 341–359.
4. Qin, A.K., Suganthan, P.N., 2005. Self-adaptive differential evolution algorithm for numerical optimization, in: 2005 IEEE Congress on Evolutionary Computation. Presented at the 2005 IEEE Congress on Evolutionary Computation, pp. 1785–1791 Vol. 2.
5. Qin, A.K., Huang, V.L., Suganthan, P.N., 2009. Differential Evolution Algorithm With Strategy Adaptation for Global Numerical Optimization. IEEE Transactions on Evolutionary Computation 13, 398–417.
6. Vrugt, J.A., Robinson, B.A., Hyman, J.M., 2009. Self-Adaptive Multimethod Search for Global Optimization in Real-Parameter Spaces. IEEE Transactions on Evolutionary Computation 13, 243–259.
7. Tanabe, R., Fukunaga, A., 2013. Success-history based parameter adaptation for Differential Evolution, in: 2013 IEEE Congress on Evolutionary Computation. Presented at the 2013 IEEE Congress on Evolutionary Computation, pp. 71–78.
8. Tanabe, R., Fukunaga, A.S., 2014. Improving the search performance of SHADE using linear population size reduction, in: 2014 IEEE Congress on Evolutionary Computation (CEC). Presented at the 2014 IEEE Congress on Evolutionary Computation (CEC), pp. 1658–1665.
9. Awad, N.H., Ali, M.Z., Suganthan, P.N., Reynolds, R.G., 2016. An ensemble sinusoidal parameter adaptation incorporated with L-SHADE for solving CEC2014 benchmark problems, in: 2016 IEEE Congress on Evolutionary Computation (CEC). Presented at the 2016 IEEE Congress on Evolutionary Computation (CEC), pp. 2958–2965.
10. Awad, N.H., Ali, M.Z., Suganthan, P.N., 2017. Ensemble sinusoidal differential covariance matrix adaptation with Euclidean neighborhood for solving CEC2017 benchmark problems, in: 2017 IEEE Congress on Evolutionary Computation (CEC). Presented at the 2017 IEEE Congress on Evolutionary Computation (CEC), pp. 372–379.
11. J.A. Vrugt, B.A. Robinson, J.M. Hyman, Self-Adaptive Multimethod Search for Global Optimization in Real-Parameter Spaces, IEEE Transactions on Evolutionary Computation. 13 (2009) 243–259. doi:10.1109/TEVC.2008.924428.
12. A.K. Qin, V.L. Huang, P.N. Suganthan, Differential Evolution Algorithm With Strategy Adaptation for Global Numerical Optimization, IEEE Transactions on Evolutionary Computation. 13 (2009) 398–417. doi:10.1109/TEVC.2008.927706.
13. R. Storn, K. Price, Differential Evolution – A Simple and Efficient Heuristic for global Optimization over Continuous Spaces, Journal of Global Optimization. 11 (1997) 341–359. doi:10.1023/A:1008202821328.
14. N.H. Awad, M.Z. Ali, P.N. Suganthan, Ensemble sinusoidal differential covariance matrix adaptation with Euclidean neighborhood for solving CEC2017 benchmark problems, in: 2017 IEEE Congress on Evolutionary Computation (CEC), 2017: pp. 372–379. doi:10.1109/CEC.2017.7969336.
15. N.H. Awad, M.Z. Ali, P.N. Suganthan, R.G. Reynolds, An ensemble sinusoidal parameter adaptation incorporated with L-SHADE for solving CEC2014 benchmark problems, in: 2016 IEEE Congress on Evolutionary Computation (CEC), 2016: pp. 2958–2965. doi:10.1109/CEC.2016.7744163.
16. Anderson, T.W., Darling, D.A., 1954. A Test of Goodness of Fit. Journal of the American Statistical Association 49, 765–769.
17. Thode, H.C., 2002. Testing For Normality. CRC Press.
18. Wilcox R., 2005. Kolmogorov–Smirnov Test. Encyclopedia of Biostatistics, Major Reference Works.
19. Zar, J.H., 2010. Biostatistical Analysis. Prentice Hall.
20. Knightes, C.D., Peters, C.A., 2000. Statistical analysis of nonlinear parameter estimation for monod biodegradation kinetics using bivariate data. Biotechnol. Bioeng. 69, 160–170.
21. Box, G.E.P., 1994. Time Series Analysis: Forecasting and Control. Prentice Hall.
22. Hamilton, J.D., 1994. Time Series Analysis. Princeton University Press.
23. Cromwell, J.B., Labys, W.C., Terraza, M., 1994. Univariate Tests for Time Series Models. SAGE.
24. Gibbons, J.D., 1996. Nonparametric Methods for Quantitative Analysis, 3rd edition. ed. Amer Sciences Pr, Columbus, Ohio.
25. Yürekli̇, K., Kurunç, A., Öztürk, F., 2005. Testing the Residuals of an ARIMA Model on the Çekerek Stream Watershed in Turkey. Turkish J Eng Env Sci 29, 61–74.
26. Engle, R.F., Lilien, D.M., Robins, R.P., 1987. Estimating Time Varying Risk Premia in the Term Structure: The Arch-M Model. Econometrica 55, 391–407.
27. Breusch, T.S., Pagan, A.R., 1979. A Simple Test for Heteroscedasticity and Random Coefficient Variation. Econometrica 47, 1287–1294.
28. White, H., 1980. A Heteroskedasticity-Consistent Covariance Matrix Estimator and a Direct Test for Heteroskedasticity. Econometrica 48, 817–838.
29. Wooldridge, J.M., 2015. Introductory Econometrics: A Modern Approach. Cengage Learning.
30. Tanabe, R., Ishibuchi, H., Oyama, A., 2017. Benchmarking Multi- and Many-Objective Evolutionary Algorithms Under Two Optimization Scenarios. IEEE Access 5, 19597–19619.
31. Igel, C., Hansen, N., Roth, S., 2007. Covariance Matrix Adaptation for Multi-objective Optimization. Evolutionary Computation 15, 1–28.
32. Deb, K., Jain, H., 2014. An Evolutionary Many-Objective Optimization Algorithm Using Reference-Point-Based Nondominated Sorting Approach, Part I: Solving Problems With Box Constraints. IEEE Transactions on Evolutionary Computation 18, 577–601.
33. Zhang, Q., Li, H., 2007. MOEA/D: A Multiobjective Evolutionary Algorithm Based on Decomposition. IEEE Transactions on Evolutionary Computation 11, 712–731.
34. Li, K., Deb, K., Zhang, Q., Kwong, S., 2015. An Evolutionary Many-Objective Optimization Algorithm Based on Dominance and Decomposition. IEEE Transactions on Evolutionary Computation 19, 694–716.
35. Li, M., Yang, S., Liu, X., 2014. Shift-Based Density Estimation for Pareto-Based Algorithms in Many-Objective Optimization. IEEE Transactions on Evolutionary Computation 18, 348–365.
36. Zitzler, E., Künzli, S., 2004. Indicator-Based Selection in Multiobjective Search, in: Parallel Problem Solving from Nature - PPSN VIII, Lecture Notes in Computer Science. Presented at the International Conference on Parallel Problem Solving from Nature, Springer, Berlin, Heidelberg, pp. 832–842.
37. Cheng, R., Jin, Y., Olhofer, M., Sendhoff, B., 2016. A Reference Vector Guided Evolutionary Algorithm for Many-Objective Optimization. IEEE Transactions on Evolutionary Computation 20, 773–791.
38. Tian, Y., Zhang, X., Cheng, R., Jin, Y., 2016. A multi-objective evolutionary algorithm based on an enhanced inverted generational distance metric, in: 2016 IEEE Congress on Evolutionary Computation (CEC). Presented at the 2016 IEEE Congress on Evolutionary Computation (CEC), pp. 5222–5229.
39. Jain, H., Deb, K., 2014. An evolutionary many-objective optimization algorithm using reference-point based nondominated sorting approach, Part II: Handling constraints and extending to an adaptive approach. IEEE Transactions on Evolutionary Computation 18, 602–622.
40. Tian, Y., Cheng, R., Zhang, X., Jin, Y., 2017. PlatEMO: A MATLAB Platform for Evolutionary Multi-Objective Optimization [Educational Forum]. IEEE Computational Intelligence Magazine 12, 73–87.
41. Zitzler, E., Deb, K., Thiele, L., 2000. Comparison of Multiobjective Evolutionary Algorithms: Empirical Results. Evolutionary Computation 8, 173–195.
42. Huband, S., Hingston, P., Barone, L., While, L., 2006. A review of multiobjective test problems and a scalable test problem toolkit. IEEE Transactions on Evolutionary Computation 10, 477–506.
43. Janssens, G.K., Pangilinan, J.M., 2010. Multiple Criteria Performance Analysis of Non-dominated Sets Obtained by Multi-objective Evolutionary Algorithms for Optimisation, in: Artificial Intelligence Applications and Innovations, IFIP Advances in Information and Communication Technology. Presented at the IFIP International Conference on Artificial Intelligence Applications and Innovations, Springer, Berlin, Heidelberg, pp. 94–103.
44. Riquelme, N., Lücken, C.V., Baran, B., 2015. Performance metrics in multi-objective optimization, in: 2015 Latin American Computing Conference (CLEI). Presented at the 2015 Latin American Computing Conference (CLEI), pp. 1–11.
45. Yen, G.G., He, Z., 2014. Performance Metric Ensemble for Multiobjective Evolutionary Algorithms. IEEE Transactions on Evolutionary Computation 18, 131–144.
46. Wang, H., Jin, Y., Yao, X., 2017. Diversity Assessment in Many-Objective Optimization. IEEE Transactions on Cybernetics 47, 1510–1522.
47. J.A. Vrugt, Multi-criteria optimization using the AMALGAM software package: Theory, concepts, and MATLAB implementation, University of California, Irvine, 2016. https://pdfs.semanticscholar.org/19cf/f034a17feda205bbc35207d83ea2c6d09074.pdf (accessed March 12, 2018).
48. M. Sadegh, J.A. Vrugt, Approximate Bayesian Computation using Markov Chain Monte Carlo simulation: DREAM(ABC), Water Resour. Res. 50 (2014) 6767–6787. doi:10.1002/2014WR015386.
49. D.B. Rubin, A. Gelman, Inference from Iterative Simulation Using Multiple Sequences, Statistical Science 7 (1992) 457-472.
50. J.A. Vrugt, Markov chain Monte Carlo simulation using the DREAM software package: Theory, concepts, and MATLAB implementation, Environmental Modelling & Software. 75 (2016) 273–316. doi:10.1016/j.envsoft.2015.08.013.
